# Country-wide expansion of a VIM-1 carbapenemase-producing *Klebsiella oxytoca* ST145 lineage in Poland, 2009–2019

**DOI:** 10.1007/s10096-023-04682-x

**Published:** 2023-10-19

**Authors:** M. Biedrzycka, P. Urbanowicz, D. Żabicka, W. Hryniewicz, M. Gniadkowski, R. Izdebski

**Affiliations:** 1 Department of Molecular Microbiology, National Medicines Institute, Chełmska 30/34, 00-725 Warsaw, Poland; 2 Department of Epidemiology and Clinical Microbiology, National Medicines Institute, Chełmska 30/34, 00-725 Warsaw, Poland

**Keywords:** VIM, Enterobacterales, *Klebsiella oxytoca* species complex, KoSC, ST145, Poland

## Abstract

**Purpose:**

To elucidate the role of the *Klebsiella oxytoca* species complex (KoSC) in epidemiology of VIM-type MBL-producing Enterobacterales in Poland.

**Methods:**

The study comprised all 106 VIM-positive KoSC isolates collected by the Polish National Reference Centre for Susceptibility Testing during 2009–2019 from 60 institutions in 35 towns. All isolates were sequenced by Illumina MiSeq, followed by MinION sequencing of selected organisms. Genomes were subjected to bioinformatic analysis, addressing taxonomy, clonality, phylogeny and structural characterisation of key resistance determinants within their chromosomal and plasmidic loci.

**Results:**

Among five species identified, *K. oxytoca* was predominant (*n* = 92), followed by *Klebsiella michiganensis* (*n* = 11). MLST distinguished 18 STs, with the most prevalent *Klebsiella oxytoca* ST145 (*n* = 83). The clone segregated a lineage with the In237-like integron [*bla*_VIM-1_–*aacA4* genes; *n* = 78], recorded in 28 cities almost all over the country. The integron was located in a ~ 49–50 kb chromosomal mosaic region with multiple other resistance genes, linked to a ~ 51 kb phage-like element. The organism might have originated from Greece, and its evolution in Poland included several events of chromosomal ~ 54–258 kb deletions, comprising the natural β-lactamase *bla*_OXY_ gene. A group of other isolates of various species and clones (*n* = 12) carried the integron In916 on self-transmissible IncA-type plasmids, effectively spreading in Italy, France and Poland.

**Conclusion:**

KoSC has been one of the major VIM producers in Poland, owing largely to clonal expansion of the specific *K. oxytoca*–In237-like lineage. Its apparently enhanced epidemic potential may create a danger on international scale.

**Supplementary Information:**

The online version contains supplementary material available at 10.1007/s10096-023-04682-x.

## Introduction

Carbapenemase-producing Enterobacterales (CPE) are considered to be largely responsible for the current antimicrobial resistance (AMR) crisis around the world [1]. One of their critically important groups is metallo-β-lactamase (MBL) producers, including those with VIM-type enzymes. Initially, these were described in *Pseudomonas* spp. in the mid-1990s, followed soon by Enterobacterales [2, 3]. In general, the VIM-type carbapenemases are encoded by gene cassettes usually of class 1 integrons that in enterobacteria are located on a variety of plasmids and only occasionally in the chromosome. The first successful VIM CPE country-wide spread in Europe was observed in Greece in the early 2000s, leading to endemic situation with numerous host species, VIM variants and their genetic determinants [4–6]. Soon, regional or interregional dissemination of such organisms has been reported in other European countries, including Spain, Italy or Hungary [6–9].

The first VIM-producing Enterobacterales isolate in Poland, *Klebsiella pneumoniae*, was confirmed in 2006 by the National Reference Centre for Susceptibility Testing (NRCST) in Warsaw [10]. Till the end of 2012, 118 VIM CPE isolates were reported, with predominance of *Enterobacter* spp. (*n* = 64; 54.2%), followed by *Klebsiella oxytoca* (*n* = 23; 19.5%) [11]. Since then, annual numbers of VIM CPE multiplied, resulting in a total of 927 isolates from 2006 to 2019 [12]. Although surpassed by *K. pneumoniae* in recent years, the *K. oxytoca* species complex (KoSC) has remained to be a highly relevant fraction of VIM CPE, ranking third among the taxonomic groups (*n* = 106; 11.4%). Here, we report the genomic analysis of all VIM-producing KoSC isolates, collected from the first identification in 2009 till the end of 2019, being part of a comprehensive WGS study of all Polish VIM CPE confirmed by the NRCST in 2006–2019.

## Materials and methods

### Bacterial isolates, WGS and species identification

The study comprised 106 non-duplicate VIM-producing KoSC isolates from 105 patients, collected by the NRCST during the CPE surveillance in Poland from 2009 to 2019 (Table S1) [12]. The isolates were from 60 centres in 35 cities of all 16 main administrative regions. The majority of the isolates were detected in the region Mazowieckie (*n* = 56; 52.8%) and often in Warsaw (*n* = 34; 32.1%). Around a half of the isolates were recovered from infections (*n* = 52; 49.1%), mainly of the urinary tract (*n* = 27; 25.5%) and wounds (*n* = 15; 14.1%), and most of the remaining ones were from carriage (*n* = 53; 50.0%). The KoSC isolates were tested for the carbapenemase presence by CARBA NP [13], phenotypic and PCR tests [14] and sequenced by MiSeq (Illumina, San Diego, CA, USA), with reads assembled with SPAdes 3.15.5 [15]. Four isolates, representing predominant *bla*_VIM_-carrying integron types, were subjected to long-read sequencing by MinION (Oxford Nanopore Technologies, Oxford, UK). The MiSeq and MinION hybrid assemblies were done with Unicycler v.0.4.8 [16]. Species identification was based on average nucleotide identity (ANI) scores, using FastANI v.1.32 with ≥ 95% cutoff [17] and RefSeq reference genomes.

### MLST, clonal and phylogenetic analyses, characterisation of* bla*_OXY_genes

MLST was performed by the mlst tool (https://github.com/tseemann/mlst). The in-sample clonality SNP analysis was done by BioNumerics v.7.6.3 (Applied Maths NV, Sint-Martens-Latem, Belgium). The SNP-based phylogenetic analysis in the international context was performed with all 271 KoSC genomes available in RefSeq as of the 1st of February 2023, using Parsnp v.1.5.4 (https://github.com/marbl/parsnp). Phylotrees were visualised by iTOL (https://itol.embl.de). Variants of the intrinsic KoSC β-lactamase *bla*_OXY_ genes were identified using the BIGSdb database (https://bigsdb.pasteur.fr/klebsiella/). Broader *bla*_OXY_-containing genomic regions were studied using the progressive Mauve algorithm [18].

### Integrons with* bla*_VIM_genes and their chromosomal and plasmid context; resistomes andantimicrobial susceptibility

The composition of *bla*_VIM_-carrying integrons was characterised manually using Geneious Prime v.2022.0.1 (Biomatters, Auckland, New Zealand) and BLASTn. Chromosomal loci containing *bla*_VIM_-carrying integrons and *bla*_OXY_ deletions were analysed using Mauve [18]; PHASTER [19] was applied to identify phage-like elements. Plasmid replicon types were identified by ABRicate using PlasmidFinder database [20]. The structural analysis of plasmids was executed using BLASTn and visualised by BRIG (http://brig.sourceforge.net/). Easyfig v.2.2.5 (http://mjsull.github.io/Easyfig/) was used to illustrate specific structures of the chromosome or plasmids with AMR genes. Acquired AMR genes (resistomes) were detected using ResFinder database, with the 99.5% identity criterion [21]. Susceptibility to 19 antimicrobials was tested for 23 representative KoSC isolates by broth microdilution, using Sensititre™ Gram Negative EUMDRXXF AST (Thermo Fisher Scientific, Waltham, MA, USA), MICRONAUT-S Pseudomonas MIC (Bruker Daltonics, Bremen, Germany), ComASP® cefiderocol (Liofilchem, Roseto degli Abruzzi, Italy) and aztreonam-avibactam in-house plates. EUCAST breakpoints (http://eucast.org) were used for interpretation of the results.

### Serotypes and virulence genes

Identification of putative virulence determinants: capsule (CPS, K) and lipopolysaccharide (LPS, O) loci and yersiniabactin and kleboxymycin biosynthesis gene clusters was performed using Kaptive [22], Kleborate [23], Geneious Prime v.2022.0.1 and BLASTn as described previously [24].

## Results

### Taxonomy, clonality and* bla*_OXY_genes

Five different KoSC species were detected among the 106 isolates: *K. oxytoca* (*n* = 92; 86.8%), *K. michiganensis* (*n* = 11, 10.4%) and *K. grimontii*, *K. pasteurii* and *K. spallanzanii* (*n* = 1; 0.9% each) (Table S1). The isolates were classified into 18 distinct STs, including five novel ones, with ten and six STs assigned to *K. oxytoca* and *K. michiganensis*, respectively (*K. spallanzanii* is not included in the MLST scheme) (Table S1). The *K. oxytoca* population was dominated vastly by ST145 (*n* = 83; 90.2% and 78.3% of *K. oxytoca* and all KoSC isolates, respectively). The occurrence of the remaining STs was marginal, including 13 STs with single isolates only.

Six phylogroups of the *bla*_OXY_ gene were congruent with the taxonomic distribution, with lineage 2 characteristic for *K. oxytoca* and lineages 1 and 5 for *K. michiganensis* (Table S1) [25, 26]. A total of 17 gene alleles were distinguished, which in general correlated well with STs. The most abundant was the *bla*_OXY-2–22_ variant (*n* = 59; 55.7% of all KoSC), observed only in the *K. oxytoca* ST145 isolates; however, a remarkable fraction of this clone (*n* = 24; 22.6% of all KoSC) were *bla*_OXY_ negative (confirmed by PCR [25]; addressed below).

### *bla*_VIM_variants and *bla*_*VIM*_*-*carrying integrons

Four *bla*_VIM_ gene variants were detected, three of which represented the *bla*_VIM-1_ lineage: *bla*_VIM-1_ (*n*=96; 90.6%), *bla*_VIM-4_ (*n*=7; 6.6%) and the novel *bla*_VIM-79_ (*n*=1; 0.9%) (Table S1). The remaining *bla*_VIM-2_ genes were sporadic (*n*=2; 1.9%). Altogether, nine *bla*_VIM_-carrying integrons were found, with predominance of In237-like elements (*bla*_VIM-1_; *n*=81, 76.4% in total), followed by In916 (*bla*_VIM-1_; *n*=13; 12.3%), and In238, In238a and In238-79 together (*bla*_VIM-4/-79_; *n*=8; 7.5% in total) (Tables S1 and S2). The In237-like integron differs from In237 by two SNPs at positions 60 and 68 in the *bla*_VIM-1_ *attC* site [11]. The In238a element differs from In238 by not having a specific 169-bp duplication at the 3′-end of the *bla*_VIM_ cassette (present also in In238-79, In237 and In237-like elements) [11, 27, 28], whereas In238-79 differs from In238 by one point mutation converting *bla*_VIM-4_to *bla*_VIM-79_.

### Epidemiology, clonality and phylogeny of the *K. oxytoca *ST145 clone

In the study period, the predominant *K. oxytoca* ST145 clone (*n* = 83) was recorded in 46 hospitals in 28 towns of 14/16 Polish administrative regions, mainly Mazowieckie (*n* = 49; 59.0% of ST145) with Warsaw (*n* = 28) (Figure S1A). The vast majority of the ST145 isolates carried the In237-like integron (*n* = 78 in total; 93.9%); few isolates had In916 or In238-like elements (*n* = 4; 4.8%, and *n* = 1; 1.2%, respectively).

**Fig. 1:**
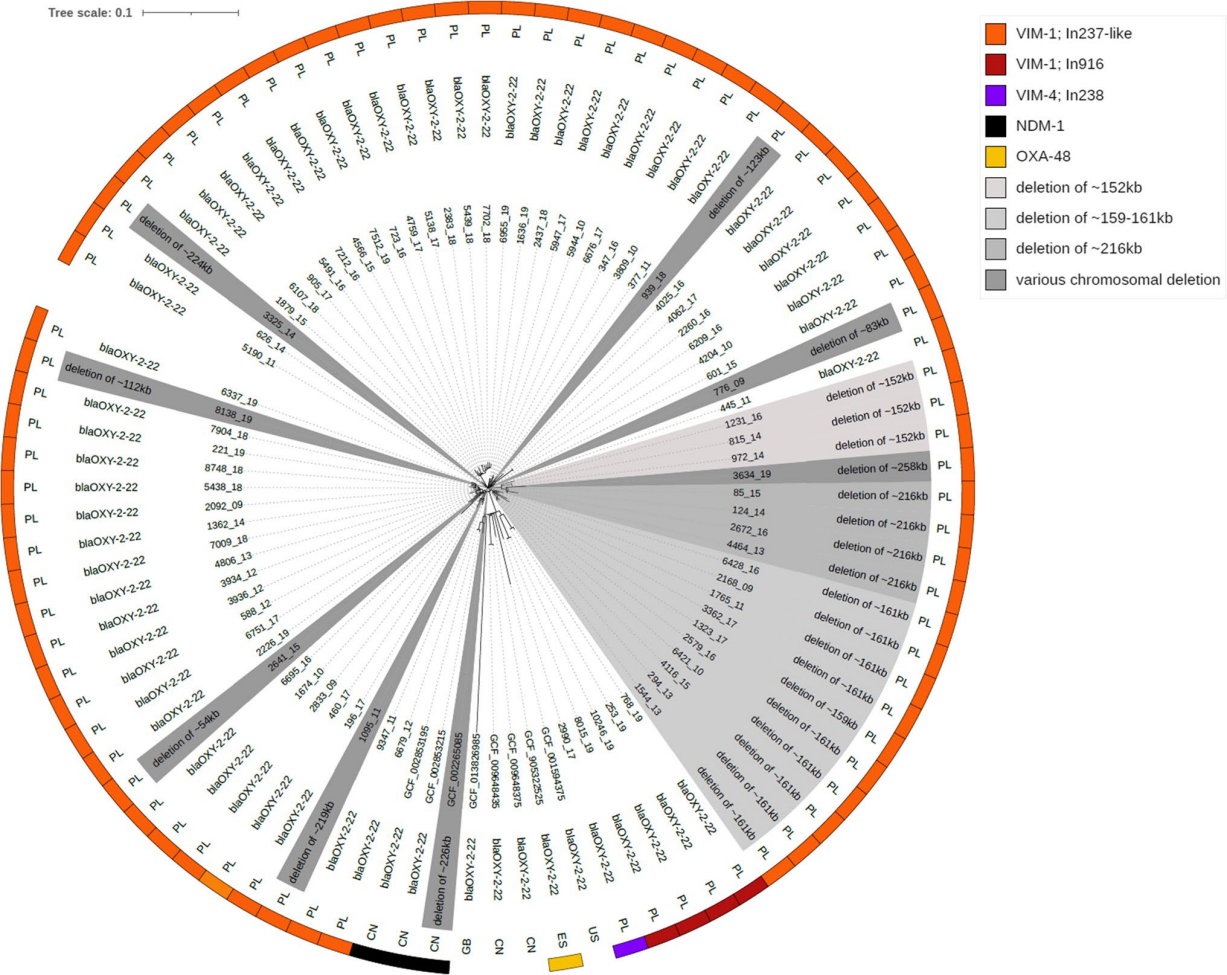
SNP-based phylogenetic tree of Polish *K. oxytoca* ST145 isolates compared with all international ST145 genomes available in RefSeq. Numbers on the inner circle are the original numbers of the study isolates or RefSeq assembly numbers. The presence of carbapenemases is indicated in the outer circles using corresponding colours. Country symbols: CN, China; ES, Spain; GB, Great Britain; PL, Poland; US, USA. The tree was constructed using Parsnp and visualised with iTOL

All of the ST145 isolates were subjected to the SNP-based clonal comparative analysis that revealed 1446 polymorphic positions within 4.9 Mb (78%) of the reference genome, characterising the original VIM-producing ST145 isolate from 2009 (isolate NMI776/09 with the In237-like integron). SNP numbers between any individual isolate and the reference ranged from 14 to 126 SNPs (Table S3). However, the 78 isolates with the In237-like element formed a distinct cluster with 0–69 SNPs between each other, indicating clonal outbreak, separated clearly from the remaining five isolates with In916 or In238 integrons (Figure S1B). The further in-depth investigation of the outbreak isolates has split them into those with the natural β-lactamase *bla*_OXY_ gene (allele *bla*_OXY-2–22_; *n* = 59) and those lacking the gene (*n* = 24). The comparison of all *bla*_OXY_-negative genomes with the ‘oldest’ ST145-In237-like *bla*_OXY_-positive isolate (isolate NMI2092/09) revealed that *bla*_OXY_ negatives have arisen from a series of chromosomal deletions, ranging from ~ 54 to ~ 258 kb (Table S4). The majority of the deletions characterised single isolates, and the phylogenetic ST145 analysis revealed these to be distributed across the phylotree, indicating mainly independent and unique character of the deletions (Fig. 1). However, some identical or similar in size deletions (~ 152 kb, ~ 159–161 kb and ~ 216 kb) were observed in multiple isolates each, and the analysis showed these to form clusters of closely related isolates, demonstrating spread of some of the *bla*_OXY_-negative sublineages, combined with further modifications of the original deletions.

The phylogenetic analysis of all 83 Polish and eight international ST145 genomes identified in RefSeq has separated the outbreak ST145-In237-like isolates from those with the In916 or In238 integrons and the isolates from other countries (China, *n* = 5; and the UK, USA and Spain, *n* = 1 each) (Fig. 1). Of note, a single isolate from China (GCF_002265085) was the only other *bla*_OXY_-negative ST145 record found in RefSeq; however, the lack of *bla*_OXY_ was observed also in isolates of ST2 from the UK (GCA_022432685) and undefined ST from South Africa (GCF_015694225).

### Phylogeny of minor STs

Only seven of the remaining STs identified in Polish KoSC were represented in the RefSeq database. A single VIM-1-positive *K. oxytoca* ST2 isolate was related to a Spanish VIM-1 producer (229 SNPs; another *bla*_VIM_ integron), and both were located on the main branch of the ST2 phylotree, along with multiple other isolates from European countries mainly (Figure S2). The other STs had only few RefSeq genomes each (results not shown).

### Acquired AMR genes and susceptibility patterns

Seventy-one acquired AMR gene profiles were defined in the study isolates, with 4–19 genes per genome and a mean of 11.1 (Table S1). The AMR gene content varied even within the ST145-In237-like outbreak cluster, presenting 46 gene profiles, each with up to 17 isolates. The only AMR genes common for all of the outbreak isolates were *bla*_VIM-1_ and *aacA4* of the In237-like integrons. Apart from other aminoglycoside resistance genes, all or most of the outbreak isolates carried the AmpC-like cephalosporinase gene *bla*_CMY-31_ (91.0%) and genes of resistance to sulphonamides (100%), trimethoprim (91.0%) and phenicols (69.2%).

Twenty-three isolates (including four ST145-In237-like outbreak organisms), representing all species, STs and various resistomes, were subjected to susceptibility testing. All these showed AMR phenotypes correlating well with the resistomes, and in general, the isolates were not extensively drug resistant (Table S5). Levels of resistance to carbapenems varied, and the majority of the isolates were susceptible or susceptible at increased exposure to meropenem (and, consistently, its combination with vaborbactam). All of the isolates had low MICs of aztreonam with avibactam, and all but one were susceptible to cefiderocol. Aminoglycosides (amikacin and gentamicin) and quinolones (especially levofloxacin) were active in vitro against the majority of the organisms, and all isolates were fully susceptible to colistin.

### Chromosomal AMR locus with the In237-like integron in the outbreak ST145 clone

**Fig. 2:**
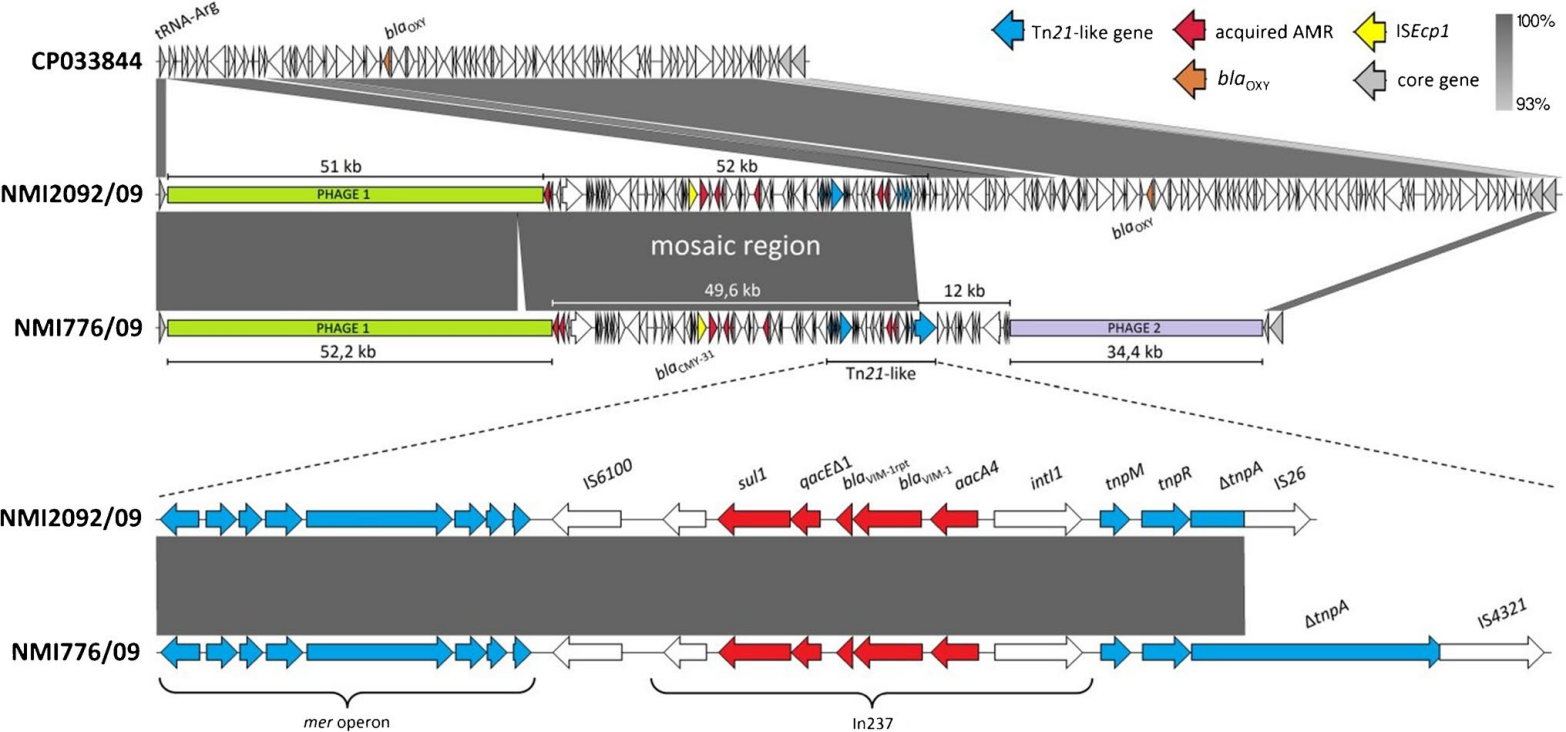
Genetic context of the chromosomal AMR islands and adjacent regions in the *bla*_OXY_-positive isolate NMI2092/09 and *bla*_OXY_-negative isolate NMI776/09, compared to the corresponding loci with tRNA^Arg^ and *bla*_OXY_ genes loci in the *K*. *oxytoca* RefSeq reference strain FDAARGOS 500 (GenBank accession number CP033844). The entire fragment compared is flanked by the tRNA^Arg^ gene (*K. oxytoca* FDAARGOS 500 locus tag EGY21_24185) and fimbrial protein genes (locus tags EGY21_24550 and EGY21_24555). Arrows indicate all CDSs proportionally to their sizes and orientation; the only selected genes or mobile elements are marked with colours and/or names. The shaded areas between linear structures indicate homologous regions and the level of their sequence identity. The Tn*21*-like structures with the In237-like integron are enlarged below the chromosomal comparisons using dotted lines

The previous, standard molecular biology study has assigned the In237-like integron to the chromosome of the early *K. oxytoca* ST145 outbreak isolates, 2009–2012 [11]. The present analysis has confirmed that observation and revealed details of the In237-like chromosomal loci. Two outbreak isolates, namely the first *bla*_OXY_-positive and *bla*_OXY_-negative isolates NMI2092/09 and NMI776/09, respectively, used as references in the clonal analyses described above, were long-read sequenced. The examination of the NMI2092/09 isolate has shown the In237-like integron to reside in a Tn*21*-like mercury resistance transposon, truncated by IS*26* and located within a unique ~ 52 kb mosaic region (MR) with multiple other mobile elements (Fig. 2). The MR contained also eight additional AMR genes (Table S1), including *bla*_CMY-31_ within an IS*Ecp1* transposition module, and it followed directly a ~ 51 kb phage-like segment, ‘Phage 1’, identified by PHASTER to be intact but of no extensive identity to any phage known. The entire ‘Phage 1’–MR structure was inserted into a tRNA^Arg^ gene with a partial, 45 bp duplication, placed ~ 29.5 kb downstream of the *bla*_OXY-2–22_ gene. The *bla*_OXY_-negative NMI776/09 isolate also contained a ‘Phage 1’–MR combo in the tRNA^Arg^ gene, sharing ~ 49.6 kb of MR with NMI2092/09, with the In237-like-Tn*21* truncated by IS*4321*. Interestingly, another phage-like structure of ~ 34 kb, ‘Phage 2’, plus additional ~ 12 kb of unknown origin was identified directly behind the MR, and the comparison with the NMI2092/09 genome revealed that this entire region has replaced ~ 83 kb of the original *K. oxytoca* chromosome, comprising *bla*_OXY_.

‘Phage 1’ was identified in all of the Polish ST145-In237-like outbreak and five ST145-In916/In238 non-outbreak isolates and in 5/8 international ST145 isolates from Spain, the USA and China, indicating no strict correlation between this structure and the MR with the In237-like-Tn*21* element. It was inserted always in the tRNA^Arg^ gene, but as revealed in two long-read sequenced non-outbreak ST145-In916/In238 isolates (NMI253/19 and NMI2990/17; described below), their ‘Phage 1’ was shorter (~ 48 kb) and had no any AMR region associated. Otherwise, apart from the NMI776/09 isolate, ‘Phage 2’ was detected in two isolates only, both *bla*_OXY_ positive, and not in any other isolate with the *bla*_OXY_ deletion.

### Plasmid profiles;* bla*_VIM_*-*carrying plasmids

At least one of 23 different plasmid replicon types was detected in 64 isolates of all STs (60.4%), producing profiles of 0–5 replicons per isolate (Table S6). The 42 isolates with no or no typeable plasmid belonged exclusively to the ST145 outbreak cluster with chromosomal In237-like elements.

Detailed structure of plasmids with *bla*_VIM_-carrying integrons was revealed for two long-read sequenced non-outbreak *K. oxytoca* ST145 isolates, having In916 (isolate NMI253/19) or In238 (NMI2990/17). In916 was identified on a ~ 134 kb IncA-type plasmid (p253A). The comparative analysis of p253A against public sequence databases showed its high identity to the previously published In916-harbouring IncA plasmids p743A, p7753A, p5955A and p9546_2 from Polish *Enterobacter* spp. or *K. pneumoniae* [12, 29], as well as a series of such plasmids from Italian Enterobacterales [30], and Dutch *Aeromonas* sp. (MH220284) (Figure S3). Main differences between all these plasmids arose from multiple rearrangements within the AMR mosaic region, containing a IS*26*–*bla*_SHV-12_–In916–IS*26* module, described originally in Italy [30]. In p253A, this region was significantly smaller than in the previously published plasmids (~ 23 kb versus ~ 37.8 to ~ 51.8 kb, respectively), had less AMR genes and no mercury resistance operon and was flanked by IS*Kpn19*-like elements on both sides (Figure S4).

In238 was located in the NMI2990/17 isolate on a ~ 23 kb plasmid (p2990) of unknown replicon. The BLASTn-based comparison revealed its low overall similarity (coverage, ~ 35%; identity, > 99%) to few international plasmids only. p2990 comprised regions encoding plasmid replication and stability, conjugal transfer (TraK, J-like and TrbJ, K, L-like families) and type I and II toxin-antitoxin systems (ptaRNA1-, RelE/StbE- and VapC-families) (results not shown).

The analysis of the short-read data for the remaining isolates with the likely plasmidic location of *bla*_VIM_ integrons was not able to associate these elements with individual plasmids in a number of these. It demonstrated In916 to reside on IncA-type plasmids in all other isolates with this integron (*n* = 12), regardless the species and ST (Table S6). The only other case was the *K. oxytoca* ST2 isolate with In238-79 (*bla*_VIM-79_) which was assigned to an IncM1-like plasmid.

### Serotypes and virulence genes

The CPS K-antigen biosynthesis locus was identified sporadically (*n* = 5, 4.7%), whereas that of the LPS O-antigen was common (*n* = 104; 98.1%), with four variants detected (Table S6). The entire *K. oxytoca* ST145 clone and single ST2, ST346 and ST347 isolates were characterised by OL104. The yersiniabactin locus was observed broadly (*n* = 94; 88.7%), including all *K. oxytoca* organisms (Table S6). The kleboxymycin biosynthesis gene cluster was detected less frequently (*n* = 40; 37.7%), being present in various genotypes, including 27 K*. oxytoca* ST145-In237-like outbreak isolates (Table S6).

## Discussion

KoSC has been an important producer of VIM-like MBLs in Poland, occupying the third position among all VIM CPE from 2006 to 2019 (11.3%), behind *Enterobacter* spp. (40.1%) and the *K. pneumoniae* species complex, KpSC (23.1%) [12]. The VIM-positive KoSC population has been mainly *K. oxytoca* itself (86.8%), which then to a similar extent has been dominated by the ST145-In237-like lineage (84.8%). Its clonal expansion has been one of the most spectacular phenomena in epidemiology of VIM CPE in Poland so far.

Originally recorded in 2009, subsequently, the *K. oxytoca* ST145-In237-like genotype has spread first in three provinces, mainly the central Mazowieckie with Warsaw [11], and then almost all over the country. It has had several specific characteristics, rarely or not observed in other Polish VIM CPE. The In237-like integron is a member of the In238 type, multiple variants of which have been identified in Enterobacterales and other Gram-negative rods in Poland and mid-Southern Europe since the 1990/2000s [11, 27, 28, 31, 32]. This individual element had been found originally in *Escherichia coli* from 2001 in Greece, the first VIM CPE ever reported [27], and in Poland, it has been recorded since 2009 mainly in the *K. oxytoca* ST145 outbreak lineage so far [11, 12, 32]. The second distinct feature of the genotype has been the location of the integron with its Tn*21*-like transposon inside the chromosomal MR linked to the putative phage, ‘Phage 1’. ‘Phage 1’ has been common in ST145 genomes, including those having no MRs with In237-like integrons, and both the scenario and mechanisms of acquisition of these individual elements remain unclear. Moreover, apart from the In237-like cassettes, the MR carried up to nine other AMR genes, with the uncommon *bla*_CMY-31_, conferring multi-drug resistance (MDR) altogether. This single chromosomal island was the major or even the only AMR source in the outbreak isolates which is rare in Enterobacterales, exploiting plasmids as main AMR genetic platforms. Overall, the isolates showed similarity to VIM-1 + CMY-31-producing *K. oxytoca* isolates from community-onset infections in Greece from 2005 to 2007, including the identical *bla*_OXY_ allele [33]. This indicated the likely origin of the ST145-In237-like lineage; however, the Greek strains have not been typed by MLST, and their genomes seem to have not been sequenced or available.

The other interesting observation regarding the epidemic ST145-In237-like genotype referred to the frequent chromosomal deletions containing *bla*_OXY_ genes, observed in 30.8% of the outbreak isolates. The *bla*_OXY_ genes encode intrinsic KoSC-specific β-lactamases, estimated to have evolved along with individual phylogenetic lineages over approximately 100 million years [25, 26]. The *bla*_OXY_ deletions have been found only in three other KoSC genomes deposited in the international databases, including one ST145 from China. Therefore, it is possible that these might have been occurring preferentially in the ST145 genetic background, which is supported also by multiplicity of independent deletion events in the study isolates. The detailed structural analysis of the index *bla*_OXY_-negative ST145-In237-like isolate suggested that the deletion could have been caused by the other putative phage (‘Phage 2’), inserted in the neighbourhood of the ‘Phage 1’–MR (In237-like) locus, and that there might have been an association between various rearrangements in this peculiar part of the genome. However, the lack of ‘Phage 2’ in other *bla*_OXY_-negative isolates excluded the hypothesis of a single deletion mechanism and has left the phenomenon of the repetitive deletions without explanation.

The second, though less important factor of the *bla*_VIM_ genes’ spread in the Polish KoSC population were In916-carrying IncA-type plasmids, observed in four non-outbreak ST145 isolates and nine isolates of various species/STs, documenting horizontal transmission. In contrast to other European countries where In916 has been associated with several plasmid incompatibility groups such as IncA, IncFII_K_, IncHI2 or IncN [34–36], in Poland, the integron has been found exclusively on IncA plasmids so far. These have been of high identity rates to those analysed in Italy [12, 29], indicating the actual origin and large-scale, successful expansion of these molecules among Enterobacterales in Europe.

In contrast to KpSC, the knowledge on KoSC virulence factors and their genetic determinants has been scarce so far. Reports on those have been usually based on homologous sequences, being extrapolations of the KpSC data, and non-including evidence from functional studies [24, 37]. Similarly, the study isolates have been checked only for the presence of several putative virulence determinants. Like in other recent studies [24, 38], the CPS K-antigen locus was of low incidence (~ 7.3%), in contrast to the LPS O-antigen biosynthesis locus (~ 97.2%) and the siderophore yersiniabactin *ybt* loci (88.7%). However, despite the high nucleotide sequence identity to the KpSC *ybt* loci, those in KoSC had different genetic context and no identifiable mobility-associated genes [39]. The gene cluster of the best-known KoSC-specific virulence factor, i.e. the toxin kleboxymycin, involved in the antibiotic-associated haemorrhagic colitis (AAHC) [40–42], was recorded in ~ 42% of the isolates. These were mainly *K. oxytoca* but also *K. michiganensis*, *K. grimontii* and *K. pasteurii*, and similar to the observations of Long et al., the cluster was absent in *K. spallanzanii*, possibly not causing the AAHC [38].

Although limited to the single country, this work has provided a remarkable amount of broader scale relevant data on epidemiology and genetics of KoSC, especially *K. oxytoca*, which has rarely been an object of specific studies. *K. oxytoca* has been a significant opportunistic pathogen and reservoir of AMR genes, and in Polish hospitals, it has been one of the most important producers of VIM-type carbapenemases. The critical part of our study concerned the *K. oxytoca* ST145-In237-like outbreak lineage of the most likely Greek origin, which since 2009 has been disseminating over the almost entire Poland’s territory. The detailed genomic analysis has revealed a number of specific characteristics of the organism, including the chromosomal MDR island carrying the In237-like integron and the *bla*_CMY-31_ cephalosporinase gene. The epidemic potential of the lineage creates a risk of its further expansion into other regions in Europe.

### Supplementary Information

Below is the link to the electronic supplementary material.

Supplementary file1 (DOCX 1298 KB)

## Data Availability

Genomic sequences have been deposited in the NCBI under the BioProject number PRJNA983967, https://www.ncbi.nlm.nih.gov/bioproject/PRJNA983967, and BioSample numbers SAMN35742457-35,742,562. Plasmid sequences have been assigned the following accession numbers: p253A, OR232699 and p2990, OR232700.
