# Supplementary material for: Country-wide expansion of a VIM-1 carbapenemase-producing Klebsiella oxytoca ST145 lineage in Poland, 2009–2019
Source: Eur J Clin Microbiol Infect Dis. 2023 Oct 19;42(12):1449–57. doi: 10.1007/s10096-023-04682-x (PMC10651708; doi:10.1007/s10096-023-04682-x)
Supplement: Supplementary file 1 — Supplementary file1 (DOCX 1298 KB) [file 10096_2023_4682_MOESM1_ESM.docx]

Country-wide expansion of a VIM-1 carbapenemase-producing *Klebsiella oxytoca* ST145 lineage in Poland, 2009-2019

M. Biedrzycka,^1^ P. Urbanowicz,^1^ D. Żabicka,^2^ W. Hryniewicz,^2^ M. Gniadkowski,^1^ and R. Izdebski^1*^

^1^*Department of Molecular Microbiology, National Medicines Institute, Chełmska 30/34, 00-725 Warsaw, Poland*

^2^*Department of Epidemiology and Clinical Microbiology, National Medicines Institute, Chełmska 30/34, 00-725 Warsaw, Poland*

* Corresponding author: Radosław Izdebski, r.izdebski@nil.gov.pl

**Table S1.** Taxonomy, clonality, *bla*_OXY_ gene variants and acquired AMR genes in the study VIM-producing KoSC isolates, 2009-2019

| Species | Isolate*^a^* | *bla*_OXY_ | ST*^b^* | *bla*_VIM_-carrying integron*^c^* | hospital | acquired AMR genes | | | | | | | | | |
| --- | --- | --- | --- | --- | --- | --- | --- | --- | --- | --- | --- | --- | --- | --- | --- |
|  |  |  |  |  |  | β-lactams*^d^* | aminoglycosides | colistin | fluoroquinolones | macrolide | phenicol | rifampicin | sulphonamide | tetracycline | trimethoprim |
| *K. oxytoca* | **776/09** | - | 145 | In237-like | Warsaw I | ***bla*_VIM-1_*,*** *bla*_CTX-M-3_*,* ***bla*_CMY-31_***, bla*_TEM-1_ | *aac(3)-IId,* ***aacA4, aph(3')-Ia, strA, strB, aadA1****, aadA2, armA* | - | *-* | *msr(E)* | ***catA1*** | *-* | ***sul1*(x2)**, *sul1* | *-* | ***dfrA1****, dfrA12* |
|  | **2092/09** | 2-22 | 145 | In237-like | Radom I | ***bla*_VIM-1_*, bla*_CMY-31_** | ***aacA4, aph(3')-Ia, strA, strB, aadA1*** | - | *-* | *-* | *-* | *-* | ***sul1(x2)*** | *-* | ***dfrA1*** |
|  | 2168/09 | - | 145 | In237-like | Warsaw VII | *bla*_VIM-1_*, bla*_CTX-M-3_*, bla*_CMY-31_*, bla*_TEM-1_ | *aac(3)-IId, aacA4, aph(3')-Ia, strA, strB, armA, aadA1, aadA2-like* | - | *-* | *mph(E), msr(E)* | *-* | *-* | *sul1* | *-* | *dfrA12* |
|  | 2833/09 | 2-22 | 145 | In237-like | Ziel. Góra | *bla*_VIM-1_*, bla*_CMY-31_ | *aacA4, aph(3')-Ia, strA, strB, aadA1* | - | *-* | *-* | *-* | *-* | *sul1* | *-* | *-* |
|  | 1674/10 | 2-22 | 145 | In237-like | Nowa Sól | *bla*_VIM-1_*, bla*_CTX-M-3_*, bla*_CMY-31_ | *aacA4, aph(3')-Ia, strA strB, armA, aadA1, aadA2* | - | *-* | *msr(E), mph(E)* | *-* | *-* | *sul1* | *-* | *dfrA1, dfrA12* |
|  | 3809/10 | 2-22 | 145 | In237-like | Warsaw XIII | *bla*_VIM-1_*, bla*_CMY-31_ | *aacA4, strA, strB, aadA1,* | - | *-* | *-* | *catA1* | *-* | *sul1* | *-* | *dfrA1* |
|  | 4204/10 | 2-22 | 145 | In237-like | Warsaw I | *bla*_VIM-1_*,* ***bla*_CMY-177_**, *bla*_DHA-1_ | *aacA4, aph(3')-Ia, strA, strB, aadA1* | - | *qnrB4* | *-* | *catA1* | *arr-3* | *sul1* | *-* | *dfrA1, dfrA27* |
|  | 5944/10 | 2-22 | 145 | In237-like | Mińsk Maz. | *bla*_VIM-1_*, bla*_CMY-31_ | *aacA4, aph(3')-Ia, strA, strB, aadA1* | - | *-* | *-* | *catA1* | *-* | *sul1* | *-* | *dfrA1* |
|  | 6421/10 | - | 145 | In237-like | Lublin III | *bla*_VIM-1_*, bla*_CMY-31_ | *aacA4, aph(3')-Ia, aadA1* | - | *-* | *-* | *catA1* | *-* | *sul1* | *-* | *dfrA1* |
|  | 377/11 | 2-22 | 145 | In237-like | Otwock | *bla*_VIM-1_*, bla*_CMY-31_ | *aacA4, aph(3')-Ia, strA, strB* | - | *-* | *-* | *catA1* | *-* | *sul1* | *-* | *dfrA1* |
|  | 445/11 | 2-22 | 145 | In237-like | Warsaw I | *bla*_VIM-1_*, bla*_CMY-31_ | *aacA4, aph(3')-Ia, strA, strB, aadA1* | - | *-* | *-* | *catA1* | *-* | *sul1* | *-* | *dfrA1* |
|  | 1095/11 | - | 145 | In237-like | Warsaw I | *bla*_VIM-1_*, bla*_CMY-31_, *bla*_DHA-1_ | *aacA4, aph(3')-Ia, strA, strB, aadA1* | - | *qnrB4* | *-* | *catA1* | *arr-3* | *sul1* | *-* | *dfrA1, dfrA27* |
|  | 1765/11 | - | 145 | In237-like | Warsaw VII | *bla*_VIM-1_*, bla*_CTX-M-15_*,bla*_OXA-1_ | *aac(3’)-IIa, aacA4, aadA1* | - | *qnrB1* | *-* | *catA1* | *-* | *sul1* | *tet(A)* | *dfrA14* |
|  | 5190/11 | 2-22 | 145 | In237-like | Warsaw I | *bla*_VIM-1_*, bla*_CMY-31_ | *aacA4, aph(3')-Ia, strA, strB, aadA1* | - | *-* | *-* | *catA1* | *-* | *sul1* | *-* | *dfrA1* |
|  | 9347/11 | 2-22 | 145 | In237-like | Warsaw I | *bla*_VIM-1_*, bla*_CMY-31_ | *aacA4, aph(3')-Ia, strA, strB, aadA1* | - | *-* | *-* | *catA1* | *-* | *sul1* | *-* | *dfrA1* |
|  | 588/12 | 2-22 | 145 | In237-like | Kostrzyn n.O. | *bla*_VIM-1_*, bla*_CMY-31_ | *aacA4, aph(3')-Ia, strA, strB, aadA1* | - | *-* | *-* | *-* | *-* | *sul1* | *-* | *dfrA1* |
|  | 3934/12 | 2-22 | 145 | In237-like | Ziel. Góra | *bla*_VIM-1_*, bla*_CMY-31_ | *aacA4, aph(3')-Ia, strA, strB, aadA1* | - | *-* | *-* | *-* | *-* | *sul1* | *-* | *dfrA1* |
|  | 3936/12 | 2-22 | 145 | In237-like | Ziel. Góra | *bla*_VIM-1_*, bla*_CMY-31_ | *aacA4, aph(3')-Ia, strA, strB, aadA1* | - | *-* | *-* | *-* | *-* | *sul1* | *-* | *dfrA1* |
|  | 6679/12 | 2-22 | 145 | In237-like | Warsaw I | *bla*_VIM-1_*, bla*_CMY-31_, *bla*_OXA-9_*, bla*_OXA-10_*, bla*_TEM-29_ | *aacA4, strA, strB* | - | *-* | *-* | *catA1* | *-* | *sul1* | *-* | *dfrA1* |
|  | 294/13 | - | 145 | In237-like | Poznań II | *bla*_VIM-1_*, bla*_CMY-31_ | *aacA4, aph(3')-Ia, strA, strB, aadA1* | - | *-* | *-* | *catA1* | *-* | *sul1* | *-* | *dfrA1* |
|  | 1544/13 | - | 145 | In237-like | Ziel. Góra | *bla*_VIM-1_*, bla*_CMY-31_ | *aacA4, aph(3')-Ia, strA, strB,aadA1* | - | *-* | *-* | *catA1* | *-* | *sul1* | *-* | *dfrA1* |
|  | 4464/13 | - | 145 | In237-like | Katowice | *bla*_VIM-1_*, bla*_CMY-31_ | *aacA4, aph(3')-Ia, aadA1* | - | *-* | *-* | *catA1* | *-* | *sul1* | *-* | *dfrA1* |
|  | 4806/13 | 2-22 | 145 | In237-like | Warsaw I | *bla*_VIM-1_ | *aacA4* | - | *-* | *-* | *-* | *-* | *sul1* | *-* | *-* |
|  | 124/14 | - | 145 | In237-like | Katowice | *bla*_VIM-1_*,bla*_CTX-M-3_*,bla*_CMY-31_*, bla*_TEM-1_, *bla*_OXA-1_ | *aac(3)-IId, aacA4, aph(3')-Ia, armA, aadA1* | - | *-* | *msr(E), mph(E)* | *catA1, catB3* | *arr-3* | *sul1* | *-* | *dfrA1* |
|  | 626/14 | 2-22 | 145 | In237-like | Osrtołęka I | *bla*_VIM-1_*, bla*_CMY-31_ | *aacA4, strA, strB, aadA1* | - | *-* | *-* | *-* | *-* | *sul1* | *-* | *dfrA1* |
|  | 815/14 | - | 145 | In237-like | Wrocław III | *bla*_VIM-1_*, bla*_CMY-31_, *bla*_CARB-2_ | *aacA4, aph(3')-Ia, strA, aphA6, strB, aadA1* | - | *-* | *-* | *catA1* | *-* | *sul1* | *-* | *dfrA1* |
|  | 972/14 | - | 145 | In237-like | Wrocław III | *bla*_VIM-1_*, bla*_CMY-31_, *bla*_CARB-2_ | *aacA4,aph(3')-Ia, strA, aphA6, strB, aadA1* | - | *-* | *-* | *catA1* | *-* | *sul1* | *-* | *dfrA1* |
|  | 1362/14 | 2-22 | 145 | In237-like | Kielce | *bla*_VIM-1_*, bla*_CMY-31_ | *aacA4, aph(3')-Ia, strA, strB, aadA1* | - | *-* | *-* | *-* | *-* | *sul1, sul2* | *-* | *dfrA1* |
|  | 3325/14 | - | 145 | In237-like | Sadowne | *bla*_VIM-1_*, bla*_CMY-31_ | *aacA4, strA, strB, aadA1* | - | *-* | *-* | *-* | *-* | *sul1* | *-* | *dfrA1* |
|  | 85/15 | - | 145 | In237-like | Katowice | *bla*_VIM-1_*, bla*_CMY-31_ | *aacA4, aph(3')-Ia, aadA1* | - | *-* | *-* | *catA1* | *-* | *sul1* | *-* | *dfrA1* |
|  | 601/15 | 2-22 | 145 | In237-like | Warsaw XV | *bla*_VIM-1_*, bla*_CMY-31_ | *aacA4, aph(3')-Ia, strA, strB, aadA1* | - | *-* | *-* | *catA1* | *-* | *sul1* | *-* | *dfrA1* |
|  | 1879/15 | 2-22 | 145 | In237-like | Ostrołęka I | *bla*_VIM-1_*, bla*_CMY-31_ | *aacA4, strA, strB, aadA1* | - | *-* | *-* | *-* | *-* | *sul1* | *-* | *dfrA1* |
|  | 2641/15 | - | 145 | In237-like | Żary | *bla*_VIM-1_*, bla*_CMY-31_*, bla*_OXA-1_ | *aacA4, aph(3’)-Ia, strA, strB, aadA1* | - | *-* | *mph(A)* | *catB3* | *arr-3* | *sul1* | *tet(A)* | *dfrA1* |
|  | 4116/15 | - | 145 | In237-like | Poznań II | *bla*_VIM-1_*, bla*_CMY-31_ | *aacA4, aph(3')-Ia, strB, aadA1* | - | *-* | *-* | *catA1* | *-* | *sul1* | *-* | *-* |
|  | 4566/15 | 2-22 | 145 | In237-like | Warsaw IX | *bla*_VIM-1_*, bla*_CMY-31_ | *aacA4, aph(3')-Ia, strA, strB, aadA1* | - | *-* | *-* | *catA1* | *-* | *sul1* | *-* | *dfrA1* |
|  | 347/16 | 2-22 | 145 | In237-like | Warsaw V | *bla*_VIM-1_*, bla*_CMY-31_ | *aacA4, strA* (x2)*, strB, aadA1* | - | *-* | *-* | *catA1* | *-* | *sul1* | *-* | *dfrA1* |
|  | 723/16 | 2-22 | 145 | In237-like | Grodzisk Maz. | *bla*_VIM-1_*, bla*_CMY-31_ | *aacA4, strA, strB, aadA1* | - | *-* | *-* | *catA1* | *-* | *sul1* | *-* | *-* |
|  | 1231/16 | - | 145 | In237-like | Grudziądz | *bla*_VIM-1_*, bla*_CTX-M-3_*, bla*_CMY-31_*, bla*_TEM-1_ | *aacA4, aph(3')-Ia, strA, strB, aadA1, armA* | - | *-* | *msr(E), mph(E)* | *catA1, catB3* | *-* | *sul1* | *-* | *dfrA1* |
|  | 2260/16 | 2-22 | 145 | In237-like | Warsaw III | *bla*_VIM-1_*, bla*_CMY-31_ | *aacA4, aph(3')-Ia, strA, aadA1* | - | *-* | *-* | *catA1* | *-* | *sul1* | *-* | *dfrA1* |
|  | 2579/16 | - | 145 | In237-like | Warsaw III | *bla*_VIM-1_*, bla*_CMY-31_ | *aacA4, aph(3')-Ia, strA, strB, aadA1* | - | *-* | *-* | *catA1* | *-* | *sul1* | *-* | *dfrA1* |
|  | 2672/16 | - | 145 | In237-like | Cieszyn | *bla*_VIM-1_*, bla*_CMY-31_ | *aacA4, aph(3')-Ia, aadA1* | - | *-* | *-* | *catA1* | *-* | *sul1* | *-* | *dfrA1* |
|  | 4025/16 | 2-22 | 145 | In237-like | Warsaw VII | *bla*_VIM-1_*, bla*_CMY-31_*, bla*_OXA-1_ | *acc(3)-IIa, aacA4, aph(3')-Ia, aadA1* | - | *-* | *-* | *catA1* | *-* | *sul1* | *-* | *dfrA1* |
|  | 5491/16 | 2-22 | 145 | In237-like | Warsaw IX | *bla*_VIM-1_*, bla*_CTX-M-3_*, bla*_CMY-31_*, bla*_TEM-1_ | *aac(3)-IId, aacA4, aph(3')-Ia, strA, strB, aadA1* | - | *qnrB19* | *-* | *catA1* | *-* | *sul1* | *-* | *dfrA1* |
|  | 6209/16 | 2-22 | 145 | In237-like | Rudka | *bla*_VIM-1_*, bla*_CMY-31_ | *aacA4, aph(3')-Ia, strA, strB, adA1* | - | *-* | *-* | *catA1* | *-* | *sul1* | *-* | *dfrA1* |
|  | 6428/16 | - | 145 | In237-like | Wołomin | *bla*_VIM-1_*, bla*_CMY-31_ | *aacA4, aph(3')-Ia, strA, strB, aadA1* | - | *-* | *-* | *catA1* | *-* | *sul1* | *-* | *dfrA1* |
|  | 6695/16 | 2-22 | 145 | In237-like | Przeworsk | *bla*_VIM-1_*, bla*_CTX-M-3_*, bla*_CMY-170_*, bla*_TEM-1_ | *aac(3)-IId, aacA4* | - | *-* | *-* | *-* | *-* | *sul1* | *-* | *-* |
|  | 7212/16 | 2-22 | 145 | In237-like | Grodzisk Maz. | *bla*_VIM-1_*, bla*_CMY-31_ | *aacA4, aph(3')-Ia, strA, strB, aadA1* | - | *-* | *-* | *catA1* | *-* | *sul1* | *-* | *dfrA1* |
|  | 196/17 | 2-22 | 145 | In237-like | Wrocław IV | *bla*_VIM-1_*, bla*_CTX-M-3_*, bla*_CMY-31_*, bla*_TEM-1_ | *aacA4, aph(3')-Ia, strA, strB, aadA1* | - | *-* | *-* | *catA1* | *-* | *sul1* | *-* | *dfrA1* |
|  | 460/17 | 2-22 | 145 | In237-like | Białystok I | *bla*_VIM-1_*, bla*_CMY-31_ | *aacA4, aph(3')-Ia, strA, strB, aadA1* | - | *-* | *-* | *catA1* | *-* | *sul1* | *-* | *dfrA1* |
|  | 905/17 | 2-22 | 145 | In237-like | Warsaw VII | *bla*_VIM-1_*, bla*_CMY-31_ | *aacA4, strA, strB, aadA1* | - | *-* | *-* | *-* | *-* | *sul1* | *-* | *dfrA1* |
|  | 1323/17 | - | 145 | In237-like | Szczecin | *bla*_VIM-1_*, bla*_CMY-31_ | *aacA4, aph(3')-Ia, strA, strB, aadA1* | - | *-* | *-* | *catA1* | *-* | *sul1* | *-* | *dfrA1* |
|  | 3362/17 | - | 145 | In237-like | Zamość | *bla*_VIM-1_*, bla*_CTX-M-3_*, bla*_CMY-31_*, bla*_TEM-1_ | *aac(3)-IId, aacA4, aph(3')-Ia, strA, strB, armA, aadA1, aadA2* | - | *-* | *mph(E), msr(E)* | *catA1* | *-* | *sul1* | *-* | *dfrA1, dfrA12* |
|  | 4062/17 | 2-22 | 145 | In237-like | Warsaw III | *bla*_VIM-1_*, bla*_CMY-31_, *bla*_OXA-1_ | *aacA4, acc(3)-IIa, aph(3')-Ia, aadA1* | - | *qnrB1, qnrS2* | *-* | *catA1* | *-* | *sul1* | *tet(A)* | *dfrA1, dfrA14* |
|  | 4759/17 | 2-22 | 145 | In237-like | Wolomin | *bla*_VIM-1_*, bla*_CMY-31,_  *bla*_OXA-1_ | *aacA4, aph(3')-Ia, strA, strB, aadA1* | - | *-* | *-* | *catA1* | *-* | *sul1* | *-* | *dfrA1* |
|  | 4793/17 | 2-22 | 145 | In237-like | Warsaw I | *bla*_VIM-1_*, bla*_KPC-2_*, bla*_TEM-1_ | *aacA4* | - | *-* | *-* | *catA1* | *-* | *sul1* | *-* | *-* |
|  | 4794/17 | 2-22 | 145 | In237-like | Warsaw I | *bla*_VIM-1_*, bla*_KPC-2_*, bla*_TEM-1_ | *aacA4* | - | *-* | *-* | *catA1* | *-* | *sul1* | *-* | *-* |
|  | 5138/17 | 2-22 | 145 | In237-like | Majdan | *bla*_VIM-1_*, bla*_CMY-31_ | *aacA4, aph(3')-Ia, strA, strB, aadA1* | - | *-* | *-* | *catA1* | *-* | *sul1* | *-* | *dfrA1* |
|  | 5947/17 | 2-22 | 145 | In237-like | Limanowa | *bla*_VIM-1_*, bla*_CTX-M-3_*, bla*_CMY-31_*, bla*_TEM-1_ | *aacA4, aac(3)-IId, aph(3')-Ia, strA, strB, aadA1* | - | *-* | *-* | *catA1* | *-* | *sul1* | *-* | *dfrA1* |
|  | 6676/17 | 2-22 | 145 | In237-like | Warsaw IV | *bla*_VIM-1_*, bla*_CMY-31_ | *aacA4, aph(3')-Ia, strA, strB, aadA1* | - | *-* | *-* | *catA1* | *-* | *sul1* | *-* | *dfrA1* |
|  | 6751/17 | 2-22 | 145 | In237-like | Ziel. Góra | *bla*_VIM-1_*, bla*_CMY-31_ | *aacA4, aph(3')-Ia, strA, strB, aadA1* | - | *-* | *-* | *-* | *-* | *sul1* | *-* | *dfrA1* |
|  | 939/18 | - | 145 | In237-like | Warsaw XVI | *bla*_VIM-1_*, bla*_CMY-31_ | *aacA4, strA, strB, aadA1* | - | *-* | *-* | *catA1* | *-* | *sul1* | *-* | *dfrA1* |
|  | 2383/18 | 2-22 | 145 | In237-like | Opole | *bla*_VIM-1_*, bla*_CMY-31_ | *aacA4, aph(3')-Ia, strA, strB, aadA1* | - | *-* | *-* | *catA1* | *-* | *sul1* | *-* | *dfrA1* |
|  | 2437/18 | 2-22 | 145 | In237-like | Piotrków Tryb. | *bla*_VIM-1_*, bla*_CMY-31_, *bla*_OXA-1_ | *acc(3)-IIa, aacA4, aph(3')-Ia* | - | *qnrB1* | *-* | *catA1* | *-* | *sul1* | *tet(A)* | *dfrA1, dfrA14* |
|  | 5438/18 | 2-22 | 145 | In237-like | Radom II | *bla*_VIM-1_*, bla*_CMY-31_ | *aacA4, aph(3')-Ia, strA, strB, aadA1* | - | *-* | *-* | *-* | *-* | *sul1* | *-* | *dfrA1* |
|  | 5439/18 | 2-22 | 145 | In237-like | Radom II | *bla*_VIM-1_*, bla*_CMY-31_ | *aacA4, aph(3')-Ia, strA, strB, aadA1* | - | *-* | *-* | *catA1* | *-* | *sul1* | *-* | *dfrA1* |
|  | 6107/18 | 2-22 | 145 | In237-like | Ostrołęka II | *bla*_VIM-1_*, bla*_CMY-31_ | *aacA43, strA, strB, aadA1* | - | *-* | *-* | *-* | *-* | *sul1* | *-* | *dfrA1* |
|  | 7009/18 | 2-22 | 145 | In237-like | Radom II | *bla*_VIM-1_*, bla*_CMY-31_ | *aacA4, aph(3')-Ia, aadA1* | - | *-* | *-* | *-* | *-* | *sul1* | *-* | *dfrA1* |
|  | 7702/18 | 2-22 | 145 | In237-like | Warsaw VI | *bla*_VIM-1_*, bla*_CMY-31_ | *aacA4, aph(3')-Ia, strA, strB, aadA1* | - | *-* | *-* | *catA1* | *-* | *sul1* | *-* | *dfrA1* |
|  | 7904/18 | 2-22 | 145 | In237-like | Radom I | *bla*_VIM-1_*, bla*_CMY-31_, *bla*_OXA-1_ | *aacA4, aph(3')-Ia, strA, strB, aadA1* | - | *qnrB1* | *-* | *-* | *-* | *sul1* | *tet(A)* | *dfrA1, dfrA14* |
|  | 8748/18 | 2-22 | 145 | In237-like | Radom I | *bla*_VIM-1_*, bla*_CMY-31_, *bla*_OXA-1_ | *aacA4, aph(3')-Ia, strA, strB, aadA1* | - | *qnrB1* | *-* | *-* | *-* | *sul1* | *tet(A)* | *dfrA1, dfrA14* |
|  | 221/19 | 2-22 | 145 | In237-like | Radom I | *bla*_VIM-1_*, bla*_CTX-M-15_*, bla*_CMY-31_,  *bla*_OXA-1_, *bla*_TEM-206_ | *aacA4, aph(3')-Ia, strB, aadA1* | - | *qnrB1* | *-* | *-* | *-* | *sul1, sul2* | *tet(A)* | *dfrA1, dfrA14* |
|  | **2990/17** | 2-22 | 145 | In238 | Białystok II | *bla*_VIM-4_*,****bla*_CTX-M-251_***,bla*_OXA-1_*, bla*_TEM-1_ | *aac(3)-IId, aacA4, aac(6’)-Ib-cr, aadA5* | - | *qnrS2* | *mph(A)* | *catB3* | *arr-3* | *sul1(3)* | *tet(B)* | *dfrA17* |
|  | 1636/19 | 2-22 | 145 | In237-like | Warsaw XIV | *bla*_VIM-1_*, bla*_CMY-31_ | *aacA4, aph(3')-Ia, strA, strB, aadA1* | - | *-* | *-* | *catA1* | *-* | *sul1* | *-* | *dfrA1* |
|  | 2226/19 | 2-22 | 145 | In237-like | Ziel. Góra | *bla*_VIM-1_*, bla*_CMY-31_ | *aacA4, aph(3')-Ia, strA, strB, aadA1* | - | *-* | *-* | *-* | *-* | *sul1* | *-* | *dfrA1* |
|  | 3634/19 | - | 145 | In237-like | Katowice | *bla*_VIM-1_*, bla*_CMY-31_ | *aac(6')-b, aph(3')-Ia, aadA1* | - | *-* | *-* | *catA1* | *-* | *sul1* | *-* | *dfrA1* |
|  | 6337/19 | 2-22 | 145 | In237-like | Radom II | *bla*_VIM-1_*, bla*_CTX-M-15_*, bla*_OXA-1_, *bla*_TEM-206_ | *aac(3a)-IIa, aacA4, strA, strB* | - | *qnrB1* | *-* | *-* | *-* | *sul1, sul2* | *tet(A)* | *dfrA14* |
|  | 6955/19 | 2-22 | 145 | In237-like | Warsaw VIII | *bla*_VIM-1_*, bla*_CMY-31_ | *aacA4, aph(3')-Ia, strA, strB, aadA1* | - | *-* | *-* | *catA1* | *-* | *sul1* | *-* | *dfrA1* |
|  | 7512/19 | 2-22 | 145 | In237-like | Warsaw X | *bla*_VIM-1_*, bla*_CMY-31_ | *aacA4, strA, strB, aadA1* | - | *-* | *-* | *catA1* | *-* | *sul1* | *-* | *dfrA1* |
|  | 8138/19 | - | 145 | In237-like | Radom I | *bla*_VIM-1_*, bla*_CTX-M-15_*, bla*_CMY-31_, *bla*_TEM-206_ | *aacA4, aph(3')-Ia, strB, aadA1* | - | *qnrB1* | *-* | *-* | *-* | *sul1, sul2* | *tet(A)* | *dfrA1, dfrA14* |
|  | **253/19** | 2-22 | 145 | In916 | Wrocław IV | *bla*_VIM-1_ | *aacA4, aphA15, aadA1* | - | *-* | *-* | *catB2* | *-* | *sul1* | *-* | *-* |
|  | 768/19 | 2-22 | 145 | In916 | Wrocław IV | *bla*_VIM-1_ | *aacA4, aphA15, aadA1* | - | *-* | *-* | *catB2* | *-* | *sul1* | *-* | *-* |
|  | 8015/19 | 2-22 | 145 | In916 | Wrocław I | *bla*_VIM-1_ | *aacA4, aphA15, aadA5* | - | *-* | *mph(A)* | *catB2* | *-* | *sul1* | *tet(B)* | *dfrA17* |
|  | 10246/19 | 2-22 | 145 | In916 | Wrocław II | *bla*_VIM-1_ | *aacA4 , aphA15, aadA5* | - | *qnrS1* | *mph(A)* | *catA1, catB2* | *-* | *sul1* | *tet(B)* | *dfrA17* |
|  | 1536/13 | 2-16 | 2 | **In238-79** | Lublin I | ***bla*_VIM-79_***, bla*_SHV-5_ | *aacA43, aadA1* | - | *-* | *-* | *-* | *-* | *sul1* | *tet(A)* | *dfrA1* |
|  | 7406/19 | 2-11 | 36 | In916 | Łodź II | *bla*_VIM-1_*, bla*_SHV-12_ | *aacA4, strA, aphA15, strB, aadA1* | - | *qnrS1* | *mph(A)* | *catB2* | *-* | *sul1, sul2* | *-* | *dfrA14* |
|  | 10316/19 | 2-12 | 37 | In71-like | Łódź I | *bla*_VIM-1_*, bla*_SHV-12_ | *aacA4, strA, aphA15, strB* | - | *qnrS1* | *mph(A)* | *-* | *-* | *sul1,sul2* | *-* | *dfrA14* |
|  | 5820/12 | 2-20 | 282 | In238 | Lublin I | *bla*_VIM-4_*, bla*_SHV-5_ | *aacA4, strA, strB, sat2* | - | *-* | *-* | *-* | *-* | *sul1, sul2* | *tet(A)* | *dfrA1* |
|  | 2978/13 | 2-12 | **346** | In916 | Gdańsk I | *bla*_VIM-1_*, bla*_SHV-12_ | *aacA4, aphA15, strA, strB, aadA1* | - | *qnrS1* | *mph(A)* | *catB2* | *-* | *sul1, sul2* | *-* | *dfrA14* |
|  | 5636/16 | 2-2 | **348** | In916 | Gdańsk I | *bla*_VIM-1_*, bla*_SHV-12_ | *aacA4, aphA15, strA, strB, aadA1* | - | *qnrS1* | *mph(A)* | *catB2* | *-* | *sul1, sul2* | *-* | *dfrA14* |
|  | 8275/17 | 2-6 | 285 | In916 | Warsaw XII | *bla*_VIM-1_ | *aacA4, strA, aphA15, strB, aadA1* | - | *qnrS1* | *-* | *catB2* | *-* | *sul1, sul2* | *-* | *-* |
|  | 388/18 | 2-2 | **347** | In916 | Cracow | *bla*_VIM-1_*, bla*_SHV-12_ | *aacA4, aphA15, aadA1* | - | *qnrS1* | *mph(A)* | *catB2* | *-* | *sul1* | *-* | *dfrA14* |
|  | 10340/19 | 2-10 | **345** | In238 | Lublin II | *bla*_VIM-4_*,bla*_CTX-M-15_*, bla*_TEM-1_ | *aac(3)-IId, aacA4* | - | *qnrS1* | *-* | *catA2* | *-* | *sul1* | *-* | *-* |
| *K. michiganensis* | 5810/12 | 1-1 | 146 | In238a | Warsaw II | *bla*_VIM-4_*, bla*_CTX-M-3_ | *aacA4, aph(3’)-Ia* | - | *-* | *-* | *-* | *-* | *sul1* | *tet(A)* | *-* |
|  | 6620/12 | 1-1 | 146 | In238a | Warsaw XI | *bla*_VIM-4_*, bla*_CTX-M-3_ | *aacA4* | - | *-* | *-* | *-* | *-* | *sul1* | *tet(A)* | *-* |
|  | 1752/13 | 5-6 | 310 | In41-like | Poznań I | *bla*_VIM-2_ | *aacA4* | - | *-* | *-* | *-* | *-* | *sul1* | *-* | *-* |
|  | 3664/13 | 5-6 | 310 | In41-like | Kościan | *bla*_VIM-2_ | *aacA4* | - | *-* | *-* | *-* | *-* | *sul1* | *-* | *-* |
|  | 3524/15 | 1-14 | 213 | In916 | Gdańsk I | *bla*_VIM-1_*, bla*_CTX-M-3_, *bla*_SHV-12_ | *aacA4, aph(3')-Ia, strA, aphA15, strB, aadA1* | - | *qnrS1* | *mph(A)* | *catB2* | *-* | *sul1, sul2* | *-* | *dfrA14* |
|  | 1085/17 | 5-1 | 95 | In237-like | Warsaw I | *bla*_VIM-1_*, bla*_OXA-1_ | *aacA4* | - | *qnrS2* | *mph(A)* | *catB3* | *arr-3* | *sul1* | *-* | *-* |
|  | 1086/17 | 5-1 | 95 | In237-like | Warsaw I | *bla*_VIM-1_*, bla*_OXA-1_ | *aacA4* | - | *qnrS2* | *mph(A)* | *catB3* | *arr-3* | *sul1* | *-* | *-* |
|  | 10279/19 | 5-1 | 95 | In237-like | Warsaw I | *bla*_VIM-1_ *bla*_OXA-1_ | *aacA4* | - | *qnrS2* | *-* | *catB3* | *arr-3* | *sul1* | *-* | *-* |
|  | 4445/19 | 5-9 | 210 | In238 | Olecko | *bla*_VIM-4_, *bla*_LAP-2_ | *aacA4* | - | *qnrS1, qnrB19* | *-* | *-* | *-* | *sul1* | *tet(A)* | *dfrA1* |
|  | 8545/19 | 1-13 | 180 | In916 | Elbląg | *bla*_VIM-1_*, bla*_SHV-12_, *bla*_GES-7_ | *aph(3')-Ia, strA, aphA15, strB, aadA1* | - | *qnrS2* | *mph(A)* | *catB2* | *-* | *sul1, sul2* | *-* | *dfrA14, dfrB3* |
|  | 9543/19 | 1-13 | 180 | In916 | Elbląg | *bla*_VIM-1_*, bla*_SHV-12_, *bla*_GES-7_ | *aph(3')-Ia, strA, aphA15, strB, aadA1* | - | *qnrS1, qnrS2* | *mph(A)* | *catB2* | *-* | *sul1, sul2* | *-* | *dfrA14, dfrB3* |
| *K. grimontii* | 5535/16 | 6-5 | 172 | In110 | Ciechanów | *bla*_VIM-1_*, bla*_ACC-1_ *bla*_LAP-2_*, bla*_OXA-10_ | *aacA4, aadA1, strA, strB* | *mcr-9.1* | *qnrS1* | *-* | *catA1, cmlA5* | *arr-2* | *sul1* | *-* | *dfrA14* |
| *K. pasteurii* | 6490/18 | 4-3 | 229 | In238a | Wrocław II | *bla*_VIM-4_ | *aacA4* | - | *-* | *-* | *-* | *-* | *sul1* | *-* | *-* |
| *K. spallanzanii* | 7090/18 | 3-3 | NA*^e^* | In916 | Poznań I | *bla*_VIM-1_*, bla*_SHV-12_ | *aacA4, aphA15, strB, aac(3’’)-Ib, aadA1* | - | *qnrS1* | *mph(A)* | *catB2* | *-* | *sul1, sul2* | *-* | *dfrA14* |

*^a^* – isolates selected to long-read sequencing are indicated in bold; total numbers of acquired AMR genes in these isolates are based on long-read sequencing; AMR genes indicated in bold are located within the chromosomal AMR islands.

*^b^* – new STs are indicated in bold.

*^c^* – new *bla*_VIM_-carrying integron variant is indicated in bold.

*^d^* – new *bla*_CMY_, *bla*_CTX-M_ and *bla*_VIM_ genes are indicated in bold; nucleotide sequences of new genes are available under the following GenBank accession numbers: *bla*_CMY-177_, OK217282; *bla*_CTX-M-251_, OK217281; *bla*_VIM-79_, OK217280.

*^e^* – NA, non-applicable; *K. spallanzanii* is not included in the MLST scheme.

**Table S2.** VIM-encoding class 1 integrons identified in the KoSC study isolates

| Integron type | Integron variant*^a^* | Gene cassette array | Species and STs | Country, year and species of the first identification*^b, c^* | | GenBank entry |  |
| --- | --- | --- | --- | --- | --- | --- | --- |
| with *bla*_VIM-1_-like genes | | | | | | | |
| In238 (n=89) | In237-like*^d^* (n=81) | 5’CS_*aacA4*_*bla*_VIM-1rpt__3’CS | *K. oxytoca* ST145  *K. michiganensis* ST95 | | Greece, 2001, *E. coli*  Greece, 2005, *A. baumanii*  Poland, 2009, *K. oxytoca* | AY152821  EF690695 | |
|  | **In238-79*^e^*** (n=1) | 5’CS_*aacA4*_*bla*_VIM-79__3’CS | *K. oxytoca* ST2 | | Poland, 2013, *K. oxytoca* | OR258282 | |
|  | In238 (n=5) | 5’CS_*aacA4*_*bla*_VIM-4rpt__3’CS | *K. oxytoca* ST145, ST282, ST345  *K. michiganensis* ST146, ST210 | | Poland, 1998, *P. aeruginosa*  Poland, 2008, *K. pneumoniae*  Poland, 2012, *K. michiganensis* | AJ585042/AY702100*^f^* | |
|  | In238a*^g^* (n=2) | 5’CS_*aacA4*_*bla*_VIM-4__3’CS | *K. michiganensis* ST146  *K. pasteurii* ST229 | | Poland, 2009, *E. hormaechei*  Poland, 2012, *K. michiganensis* | JQ003906 (Hungary 2010) | |
| In916 (n=13) | In916 (n=13) | 5’CS_*bla*_VIM-1__*aacA4*_*aphA15*_*aadA1*_*catB2*_3’CS | *K. oxytoca* ST36, ST145, ST285, ST346, ST347, ST348  *K. michiganensis* ST180, ST231  *K. spallanzanii* ND | | Spain, before 2014, *E. coli*  Poland, 2013, *E. coli* & *C. freundii*  Poland, 2013, *K. oxytoca* | KF856617 | |
| In70 (n=1) | In71-like*^h^* | 5’CS_*bla*_VIM-1__*aacA4*_*aphA15*_3’CS | *K. oxytoca* ST137 | | Italy, 2016, *E. cloacae*  Poland, 2019, *K. oxytoca* | AM183120 | |
| In110 (n=1) | In110 | 5’CS_*bla*_VIM-1__*aacA4*_*aadA1*_3’CS | *K. grimontii* ST172 | | Italy, 1999, *P. putida*  Poland, 2006, *P. aeruginosa*  Poland, 2016, *E. hormaechei* & *K. grimontii* | AJ439689 | |
| with *bla*_VIM-2_-like genes | | | | | | | |
| In41 (n=2) | In41-like*^i^* (n=2) | 5’CS_*bla*_VIM-2__*aacA4*_3’CS | *K. michiganensis* ST310 | Argentina, 2014, *P.putida*  Poland, 2013, *K. michiganensis* | | KF840720 |  |

*^a^* – the new integron is indicated in bold.

*^b^* – when the first report was from another country, then it is followed by the first Polish case(s); if the first Polish record was from non-Enterobacterales and/or non-KoSC, it is then followed by the first Polish Enterobacterales and KoSC, respectively.

*^c^* – date of isolation of the first Polish organism with a given integron may be earlier than that of the first isolate reported ever in another country.

*^d^* – the In237-like GenBank entry EF690695 of an *A. baumannii* isolate stands for In237 in the INTEGRALL database (www.integrall.bio.ua.pt); the enterobacterial In237-like

integrons from Greece AY152821 and Poland differ from that by two SNPs in the *bla*_VIM_ 59-be element.

*^e^* – In238-79 differs from In238 by one point mutation converting *bla*_VIM-4_ to *bla*_VIM-79_; due to temporary suspended activity of the INTEGRALL database In238-79 has not been numbered according to that.

*^f^* – the original In238 record (AJ585042) contains a 2 nt error in the *bla*_VIM-4_ coding sequence; the subsequent *P. aeruginosa* In238 entry from Hungary from 2003 has been provided.

*^g^* – In238a differs from the In238 element by having no 3’-terminal 169bp tandem repeat in the *bla*_VIM-4_ gene cassette.

*^h^* – In71-like differs from the In71 element by *bla*_VIM-1_ gene cassette instead of *bla*_VIM-1b_.

*^i^* – In41-like differs from the In41 element by having the 3’CS region.

**Table S3.** SNP scores between *K. oxytoca* ST145 isolates

| Isolate | Region | Hospital | Number of SNPs | OXY variant | Integron variant | Remarks |
| --- | --- | --- | --- | --- | --- | --- |
| 776/09*^a^* | Mazowieckie | Warsaw I | 0 | - | In237-like | ST145-In237-like-VIM-1 outbreak |
| 445/11 | Mazowieckie | Warsaw I | 14 | 2-22 | In237-like | ST145-In237-like-VIM-1 outbreak |
| 5190/11 | Mazowieckie | Warsaw I | 14 | 2-22 | In237-like | ST145-In237-like-VIM-1 outbreak |
| 601/15 | Mazowieckie | Warsaw XV | 14 | 2-22 | In237-like | ST145-In237-like-VIM-1 outbreak |
| 5944/10 | Mazowieckie | Mińsk Maz. | 16 | 2-22 | In237-like | ST145-In237-like-VIM-1 outbreak |
| 3809/10 | Mazowieckie | Warsaw XIII | 18 | 2-22 | In237-like | ST145-In237-like-VIM-1 outbreak |
| 1879/15 | Mazowieckie | Ostrołęka I | 18 | 2-22 | In237-like | ST145-In237-like-VIM-1 outbreak |
| 2833/09 | Lubuskie | Ziel. Góra | 19 | 2-22 | In237-like | ST145-In237-like-VIM-1 outbreak |
| 626/14 | Mazowieckie | Ostrołęka I | 20 | 2-22 | In237-like | ST145-In237-like-VIM-1 outbreak |
| 4204/10 | Mazowieckie | Warsaw I | 21 | 2-22 | In237-like | ST145-In237-like-VIM-1 outbreak |
| 1674/10 | Lubuskie | Nowa Sól | 23 | 2-22 | In237-like | ST145-In237-like-VIM-1 outbreak |
| 4794/17 | Mazowieckie | Warsaw I | 23 | 2-22 | In237-like | ST145-In237-like-VIM-1 outbreak |
| 2092/09 | Mazowieckie | Radom I | 26 | 2-22 | In237-like | ST145-In237-like-VIM-1 outbreak |
| 3325/14 | Mazowieckie | Sadowne | 27 | - | In237-like | ST145-In237-like-VIM-1 outbreak |
| 905/17 | Mazowieckie | Warsaw VII | 28 | 2-22 | In237-like | ST145-In237-like-VIM-1 outbreak |
| 377/11 | Mazowieckie | Otwock | 29 | 2-22 | In237-like | ST145-In237-like-VIM-1 outbreak |
| 1765/11 | Mazowieckie | Warsaw VII | 30 | - | In237-like | ST145-In237-like-VIM-1 outbreak |
| 4025/16 | Mazowieckie | Warsaw VII | 30 | 2-22 | In237-like | ST145-In237-like-VIM-1 outbreak |
| 2168/09 | Mazowieckie | Warsaw VII | 31 | - | In237-like | ST145-In237-like-VIM-1 outbreak |
| 5947/17 | Małopolskie | Limanowa | 31 | 2-22 | In237-like | ST145-In237-like-VIM-1 outbreak |
| 7009/18 | Mazowieckie | Radom II | 31 | 2-22 | In237-like | ST145-In237-like-VIM-1 outbreak |
| 2437/18 | Łódzkie | Piotrków Tryb. | 32 | 2-22 | In237-like | ST145-In237-like-VIM-1 outbreak |
| 6421/10 | Lubelskie | Lublin III | 33 | - | In237-like | ST145-In237-like-VIM-1 outbreak |
| 588/12 | Lubuskie | Kostrzyn n.O. | 33 | 2-22 | In237-like | ST145-In237-like-VIM-1 outbreak |
| 3934/12 | Lubuskie | Ziel. Góra | 33 | 2-22 | In237-like | ST145-In237-like-VIM-1 outbreak |
| 3936/12 | Lubuskie | Ziel. Góra | 33 | 2-22 | In237-like | ST145-In237-like-VIM-1 outbreak |
| 2579/16 | Mazowieckie | Warsaw III | 33 | - | In237-like | ST145-In237-like-VIM-1 outbreak |
| 1095/11 | Mazowieckie | Warsaw I | 34 | - | In237-like | ST145-In237-like-VIM-1 outbreak |
| 723/16 | Mazowieckie | Grodzisk Maz. | 34 | 2-22 | In237-like | ST145-In237-like-VIM-1 outbreak |
| 4566/15 | Mazowieckie | Warsaw IX | 35 | 2-22 | In237-like | ST145-In237-like-VIM-1 outbreak |
| 5438/18 | Mazowieckie | Radom II | 35 | 2-22 | In237-like | ST145-In237-like-VIM-1 outbreak |
| 6107/18 | Mazowieckie | Ostrołęka II | 35 | 2-22 | In237-like | ST145-In237-like-VIM-1 outbreak |
| 347/16 | Mazowieckie | Warsaw V | 36 | 2-22 | In237-like | ST145-In237-like-VIM-1 outbreak |
| 1362/14 | Świetokrzyskie | Kielce | 36 | 2-22 | In237-like | ST145-In237-like-VIM-1 outbreak |
| 294/13 | Wielkopolskie | Poznań II | 37 | - | In237-like | ST145-In237-like-VIM-1 outbreak |
| 9347/11 | Mazowieckie | Warsaw I | 39 | 2-22 | In237-like | ST145-In237-like-VIM-1 outbreak |
| 1544/13 | Lubuskie | Ziel. Góra | 39 | - | In237-like | ST145-In237-like-VIM-1 outbreak |
| 6428/16 | Mazowieckie | Wołomin | 40 | - | In237-like | ST145-In237-like-VIM-1 outbreak |
| 7212/16 | Mazowieckie | Grodzisk Maz. | 40 | 2-22 | In237-like | ST145-In237-like-VIM-1 outbreak |
| 939/18 | Mazowieckie | Warsaw XVI | 40 | - | In237-like | ST145-In237-like-VIM-1 outbreak |
| 4116/15 | Wielkopolskie | Poznań II | 41 | - | In237-like | ST145-In237-like-VIM-1 outbreak |
| 460/17 | Podlaskie | Białystok I | 41 | 2-22 | In237-like | ST145-In237-like-VIM-1 outbreak |
| 6676/17 | Mazowieckie | Warsaw IV | 41 | 2-22 | In237-like | ST145-In237-like-VIM-1 outbreak |
| 6751/17 | Lubuskie | Ziel. Góra | 41 | 2-22 | In237-like | ST145-In237-like-VIM-1 outbreak |
| 221/19 | Mazowieckie | Radom I | 41 | 2-22 | In237-like | ST145-In237-like-VIM-1 outbreak |
| 8138/19 | Mazowieckie | Radom I | 42 | - | In237-like | ST145-In237-like-VIM-1 outbreak |
| 7904/18 | Mazowieckie | Radom I | 42 | 2-22 | In237-like | ST145-In237-like-VIM-1 outbreak |
| 6337/19 | Mazowieckie | Radom II | 44 | 2-22 | In237-like | ST145-In237-like-VIM-1 outbreak |
| 6679/12 | Mazowieckie | Warsaw I | 45 | 2-22 | In237-like | ST145-In237-like-VIM-1 outbreak |
| 4806/13 | Mazowieckie | Warsaw I | 46 | 2-22 | In237-like | ST145-In237-like-VIM-1 outbreak |
| 8748/18 | Mazowieckie | Radom I | 46 | 2-22 | In237-like | ST145-In237-like-VIM-1 outbreak |
| 5138/17 | Mazowieckie | Majdan | 47 | 2-22 | In237-like | ST145-In237-like-VIM-1 outbreak |
| 4759/17 | Mazowieckie | Wolomin | 47 | 2-22 | In237-like | ST145-In237-like-VIM-1 outbreak |
| 2383/18 | Opolskie | Opole | 48 | 2-22 | In237-like | ST145-In237-like-VIM-1 outbreak |
| 5439/18 | Mazowieckie | Radom II | 49 | 2-22 | In237-like | ST145-In237-like-VIM-1 outbreak |
| 7512/19 | Mazowieckie | Warsaw X | 50 | 2-22 | In237-like | ST145-In237-like-VIM-1 outbreak |
| 4464/13 | Śląskie | Katowice | 51 | - | In237-like | ST145-In237-like-VIM-1 outbreak |
| 85/15 | Śląskie | Katowice | 52 | - | In237-like | ST145-In237-like-VIM-1 outbreak |
| 1636/19 | Mazowieckie | Warsaw XIV | 52 | 2-22 | In237-like | ST145-In237-like-VIM-1 outbreak |
| 124/14 | Śląskie | Katowice | 53 | - | In237-like | ST145-In237-like-VIM-1 outbreak |
| 2641/15 | Lubuskie | Żary | 53 | - | In237-like | ST145-In237-like-VIM-1 outbreak |
| 7702/18 | Mazowieckie | Warsaw VI | 53 | 2-22 | In237-like | ST145-In237-like-VIM-1 outbreak |
| 6955/19 | Mazowieckie | Warsaw VIII | 54 | 2-22 | In237-like | ST145-In237-like-VIM-1 outbreak |
| 2226/19 | Lubuskie | Ziel. Góra | 54 | 2-22 | In237-like | ST145-In237-like-VIM-1 outbreak |
| 3362/17 | Lubelskie | Zamość | 55 | - | In237-like | ST145-In237-like-VIM-1 outbreak |
| 196/17 | Dolnośląskie | Wrocław IV | 56 | 2-22 | In237-like | ST145-In237-like-VIM-1 outbreak |
| 4062/17 | Mazowieckie | Warsaw III | 57 | 2-22 | In237-like | ST145-In237-like-VIM-1 outbreak |
| 815/14 | Dolnośląskie | Wrocław III | 61 | - | In237-like | ST145-In237-like-VIM-1 outbreak |
| 1323/17 | Zachodniopomorskie | Szczecin | 61 | - | In237-like | ST145-In237-like-VIM-1 outbreak |
| 972/14 | Dolnośląskie | Wrocław III | 62 | - | In237-like | ST145-In237-like-VIM-1 outbreak |
| 5491/16 | Mazowieckie | Warsaw IX | 64 | 2-22 | In237-like | ST145-In237-like-VIM-1 outbreak |
| 1231/16 | Kujawsko-Pomorskie | Grudziądz | 65 | - | In237-like | ST145-In237-like-VIM-1 outbreak |
| 2672/16 | Śląskie | Cieszyn | 69 | - | In237-like | ST145-In237-like-VIM-1 outbreak |
| 6209/16 | Mazowieckie | Rudka | 69 | 2-22 | In237-like | ST145-In237-like-VIM-1 outbreak |
| 2260/16 | Mazowieckie | Warsaw III | 69 | 2-22 | In237-like | ST145-In237-like-VIM-1 outbreak |
| 6695/16 | Podkarpackie | Przeworsk | 80 | 2-22 | In237-like | ST145-In237-like-VIM-1 outbreak |
| 3634/19 | Śląskie | Katowice | 90 | - | In237-like | ST145-In237-like-VIM-1 outbreak |
| 253/19 | Dolnośląskie | Wrocław IV | 110 | 2-22 | In916 | ST145-In916-VIM-1 hospital dissemination |
| 768/19 | Dolnośląskie | Wrocław IV | 115 | 2-22 | In916 | ST145-In916-VIM-1 hospital dissemination |
| 10246/19 | Dolnośląskie | Wrocław II | 125 | 2-22 | In916 | ST145-In916-VIM-1 local dissemination |
| 8015/19 | Dolnośląskie | Wrocław I | 126 | 2-22 | In916 | ST145-In916-VIM-1 local dissemination |
| 2990/17 | Podlaskie | Białystok II | 125 | 2-22 | In238 | ST145-In238-VIM4 single case |

*^a^* – reference isolate, *i.e.* the Poland’s index isolate of ST145 as confirmed by the National Reference Centre for Susceptibility Testing.

**Table S4**. Chromosomal deletions of the *bla*_OXY_ gene regions in *K.oxytoca* ST145 *bla*_OXY_-negative isolates

| Isolate | Region | Hospital | Size of the chromosome deletion*^a^* | Group of isolates with similar size of deletion |
| --- | --- | --- | --- | --- |
| 2168/09 | Mazowieckie | Warsaw VII | ~161 kb | A |
| 6421/10 | Lubelskie | Lublin III | ~161 kb | A |
| 1765/11 | Mazowieckie | Warsaw VII | ~161 kb | A |
| 294/13 | Wielkopolskie | Poznań II | ~161 kb | A |
| 1544/13 | Lubuskie | Ziel. Góra | ~161 kb | A |
| 4116/15 | Wielkopolskie | Poznań II | ~161 kb | A |
| 2579/16 | Mazowieckie | Warsaw III | ~161 kb | A |
| 6428/16 | Mazowieckie | Wołomin | ~161 kb | A |
| 1323/17 | Zachodniopomorskie | Szczecin | ~159 kb | A |
| 3362/17 | Lubelskie | Zamość | ~161 kb | A |
| 4464/13 | Śląskie | Katowice | ~216 kb | B |
| 124/14 | Śląskie | Katowice | ~216 kb | B |
| 85/15 | Śląskie | Katowice | ~216 kb | B |
| 2672/16 | Śląskie | Cieszyn | ~216 kb | B |
| 815/14 | Dolnośląskie | Wrocław III | ~152 kb | C |
| 972/14 | Dolnośląskie | Wrocław III | ~152 kb | C |
| 1231/16 | Kujawsko-Pomorskie | Grudziądz | ~152 kb | C |
| 2641/15 | Lubuskie | Żary | ~54 kb | single |
| 776/09 | Mazowieckie | Warsaw I | 82,724 bp*^b^* | single |
| 8138/19 | Mazowieckie | Radom I | ~112 kb | single |
| 939/18 | Mazowieckie | Warsaw XVI | ~123 kb | single |
| 1095/11 | Mazowieckie | Warsaw I | ~219 kb | single |
| 3325/14 | Mazowieckie | Sadowne | ~224 kb | single |
| 3634/19 | Śląskie | Katowice | ~258 kb | single |

*^a^* – sizes of the chromosomal deletions have been estimated by the comparison of individual *bla*_OXY_-negative genomes, to the reference *bla*_OXY_-positive ST145-In237-like isolate NMI2092/09; for all isolates but NMI776/09, the sizes were estimated by the progressive Mauve algorithm, using Geneious Prime v.2022.0.1 (Biomatters, Auckland, New Zealand).

*^b^* – the precise size of the chromosome deletion in the NMI776/09 isolate was determined by the comparison of long-read sequences.

**Table S5.** MICs of antimicrobials for the KoSC isolates

| Isolates*^a^* | | MIC (mg/L)*^b,c^* | | | | | | | | | | | | | | | | | | |
| --- | --- | --- | --- | --- | --- | --- | --- | --- | --- | --- | --- | --- | --- | --- | --- | --- | --- | --- | --- | --- |
|  |  | PIP | TZP*^d^* | CAZ | FEP | ATM | IPM | MEM | CZA*^d^* | AZA*^d^* | I-R*^d^* | MVB*^d^* | FDC | AMK | GEN | TOB | CIP | LVX | SXT | CST |
| 776/09 | *Kox* ST145 | **>32** | **>32** | **>32** | **>16** | **16** | 2 | 2 | **>16** | ≤0.06 | 2 | 2 | 2 | **>32** | **>32** | **>4** | **>8** | **>8** | **>8** | 1 |
| 2092/09 | *Kox* ST145 | **>32** | **>32** | **>32** | **8** | **8** | ≤1 | 0.5 | **>16** | 0.25 | 1 | 0.25 | 0.5 | 8 | 2 | **>4** | **8** | **4** | **>8** | ≤0.5 |
| 2833/09 | *Kox* ST145 | **32** | **>32** | **>32** | **8** | *4* | 2 | 0.5 | **>16** | 0.25 | 2 | 0.25 | 0.5 | **16** | 0.5 | **>4** | **4** | **2** | **>8** | ≤0.5 |
| 2990/17 | *Kox* ST145 | **>32** | **>32** | **16** | *4* | *4* | **8** | *8* | **16** | 0.25 | **8** | 4 | 0.25 | **16** | **>32** | **>4** | **>8** | **>8** | **>8** | ≤0.5 |
| 3634/19 | *Kox* ST145 | **>32** | **>32** | **>32** | **16** | **8** | *4* | *4* | **>16** | 0.25 | **4** | 4 | 0.125 | **16** | 2 | **>4** | **>8** | **>8** | **>8** | ≤0.5 |
| 5820/12 | *Kox* ST282 | **>32** | **>32** | **>32** | *2* | **>32** | *4* | 1 | **16** | ≤0.06 | **4** | 1 | 0.5 | **16** | ≤0.25 | **>4** | ≤0.06 | ≤0.125 | **>8** | ≤0.5 |
| 1536/13 | *Kox* ST2 | **>32** | **>32** | **>32** | **8** | **>32** | **>8** | *4* | **16** | 0.5 | **>8** | 4 | 2 | **16** | ≤0.25 | **>4** | ≤0.06 | ≤0.125 | **>8** | ≤0.5 |
| 2978/13 | *Kox* ST346 | **>32** | **>32** | **>32** | **16** | **>32** | **8** | 2 | **>16** | 0.125 | **8** | 2 | 0.5 | ≤2 | 1 | **>4** | **1** | *1* | **>8** | ≤0.5 |
| 5636/16 | *Kox* ST348 | **>32** | **>32** | **>32** | **16** | **>32** | *4* | 2 | **>16** | ≤0.06 | **4** | 2 | 0.5 | ≤2 | 1 | **>4** | *0.5* | 0.5 | **>8** | ≤0.5 |
| 388/18 | *Kox* ST347 | **>32** | **>32** | **>32** | **>16** | **>32** | **8** | *8* | **>16** | ≤0.06 | **8** | 8 | 2 | ≤2 | 2 | **>4** | *0.5* | 0.5 | **>8** | ≤0.5 |
| 7406/19 | *Kox* ST36 | **>32** | **>32** | **>32** | **>16** | **>32** | **8** | *8* | **>16** | 0.25 | **8** | 8 | **4** | ≤2 | 1 | **>4** | *0.5* | 0.5 | **>8** | ≤0.5 |
| 10340/19 | *Kox* ST345 | **>32** | **32** | **16** | *4* | **32** | 2 | 0.25 | 4 | ≤0.06 | 2 | 0.25 | 0.5 | 4 | **32** | **>4** | 0.125 | 0.25 | ≤1 | ≤0.5 |
| 10316/19 | *Kox* ST37 | **>32** | **>32** | **>32** | **>16** | **32** | **8** | *4* | **>16** | ≤0.06 | **8** | 4 | 2 | ≤2 | 1 | **>4** | **1** | 0.5 | **>8** | ≤0.5 |
| 8275/17 | *Kox* ST285 | **>32** | **>32** | **>32** | **>16** | ≤1 | **8** | **16** | **>16** | 0.125 | **8** | **16** | 1 | ≤2 | 1 | **>4** | *0.5* | 0.5 | ≤1 | ≤0.5 |
| 5810/12 | *Kmi* ST146 | **>32** | **>32** | **32** | *4* | **8** | **8** | *8* | **16** | 0.125 | **8** | 4 | 0.5 | **16** | ≤0.25 | **>4** | *0.5* | 0.5 | **>8** | 1 |
| 1752/13 | *Kmi* ST310 | **32** | **32** | *4* | ≤1 | ≤1 | *4* | 0.5 | 4 | ≤0.06 | **4** | 0.5 | 0.5 | ≤2 | **4** | **>4** | ≤0.06 | ≤0.125 | ≤1 | ≤0.5 |
| 3524/15 | *Kmi* ST213 | **>32** | **>32** | **>32** | **>16** | **>32** | **8** | **16** | **>16** | 0.25 | **8** | **16** | 1 | ≤2 | 1 | **>4** | **>8** | **>8** | **>8** | ≤0.5 |
| 4445/19 | *Kmi* ST210 | **>32** | **>32** | *2* | ≤1 | ≤1 | 2 | 0.5 | 4 | 0.25 | **4** | 0.5 | 0.125 | ≤2 | 0.5 | **>4** | **4** | *1* | **>8** | ≤0.5 |
| 8545/19 | *Kmi* ST180 | **>32** | **>32** | **>32** | **16** | **32** | **8** | *4* | **>16** | ≤0.06 | **8** | 4 | 1 | ≤2 | 1 | **>4** | **1** | 0.5 | **>8** | ≤0.5 |
| 1085/17 | *Kmi* ST95 | **>32** | **>32** | **8** | *4* | ≤1 | *4* | 0.5 | **>16** | ≤0.06 | **4** | 0.5 | 1 | 8 | 0.5 | **>4** | **2** | 0.5 | ≤1 | ≤0.5 |
| 5535/16 | *Kgr* ST172 | **>32** | **>32** | **>32** | **16** | *2* | *4* | 2 | **>16** | 0.5 | **4** | 2 | 0.5 | ≤2 | 1 | **4** | **4** | **2** | **>8** | ≤0.5 |
| 6490/18 | *Kpa* ST229 | **32** | **32** | 1 | ≤1 | ≤1 | *4* | 0.5 | 4 | ≤0.06 | **4** | 0.25 | 0.125 | 4 | ≤0.25 | **>4** | ≤0.06 | ≤0.125 | ≤1 | ≤0.5 |
| 7090/18 | *Ksp* | **>32** | **>32** | **>32** | **>16** | **>32** | *4* | **16** | **>16** | 0.125 | **8** | 8 | 2 | ≤2 | 1 | **>4** | *0.5* | 0.5 | **>8** | ≤0.5 |

*^a^* – *Kox*, *K. oxytoca*; *Kmi*, *K. michiganensis*; *Kgr*, *K. grimontii*; *Kpa*, *K. pasteurii*; *Ksp*, *K. spallanzanii*

*^b^* – abbreviations: PIP, piperacillin; TZP, piperacillin-tazobactam; CAZ, ceftazidime; FEP, cefepime; ATM, aztreonam; IPM, imipenem; MEM, meropenem; CZA, ceftazidime-avibactam; AZA, aztreonam-avibactam; I-R, imipenem-relebactam; MVB, meropenem-vaborbactam; FDC, cefiderocol; AMK, amikacin; GEN, gentamicin; TOB, tobramycin; CIP, ciprofloxacin; LVX, levofloxacin; SXT, trimethoprim-sulfamethoxazole; CST, colistin.

*^c^*– bold, italic and normal styles refer to resistance, susceptibility increased exposure and susceptibility, respectively, according to EUCAST (http://eucast.org); the results for aztreonam-avibactam were not interpreted owing to the lack of criteria, however, the MICs against all of the isolates were in the category ‘susceptible’ for aztreonam alone.

*^d^* – tazobactam, avibactam, relebactam and vaborbactam were at fixed concentrations of 4, 4, 4 and 8 mg/L, respectively.

**Table S6**. Virulence loci, serotypes and plasmid replicon profiles in the study VIM-producing KoSC isolates

| Species | Isolate | ST | *bla*_VIM_ integron | Yersiniabactin | Kleboxymycin | K locus | O locus | Plasmid replicons |
| --- | --- | --- | --- | --- | --- | --- | --- | --- |
| *K. oxytoca* | 776/09 | 145 | In237-like | + | *-* | *-* | OL104 | IncM2 |
|  | 2092/09 | 145 | In237-like | *+* | *+* | *-* | OL104 | *-* |
|  | 2168/09 | 145 | In237-like | *+* | *+* | *-* | OL104 | IncM2 |
|  | 2833/09 | 145 | In237-like | + | *-* | *-* | OL104 | *-* |
|  | 1674/10 | 145 | In237-like | *+* | *+* | *-* | OL104 | IncM2 |
|  | 3809/10 | 145 | In237-like | + | *-* | *-* | OL104 | *-* |
|  | 4204/10 | 145 | In237-like | + | *-* | *-* | OL104 | *-* |
|  | 5944/10 | 145 | In237-like | + | *-* | *-* | OL104 | *-* |
|  | 6421/10 | 145 | In237-like | *+* | *+* | *-* | OL104 | *-* |
|  | 377/11 | 145 | In237-like | + | *-* | *-* | OL104 | *-* |
|  | 445/11 | 145 | In237-like | + | *-* | *-* | OL104 | *-* |
|  | 1095/11 | 145 | In237-like | *+* | *+* | *-* | OL104 | *-* |
|  | 1765/11 | 145 | In237-like | *+* | *+* | *-* | OL104 | IncHI2, IncHI2A |
|  | 5190/11 | 145 | In237-like | + | *-* | *-* | OL104 | IncFIB_K_, IncFII_K_ |
|  | 9347/11 | 145 | In237-like | + | *-* | *-* | OL104 | *-* |
|  | 588/12 | 145 | In237-like | + | *-* | *-* | OL104 | *-* |
|  | 3934/12 | 145 | In237-like | + | *-* | *-* | OL104 | *-* |
|  | 3936/12 | 145 | In237-like | + | *-* | *-* | OL104 | *-* |
|  | 6679/12 | 145 | In237-like | - | + | *-* | OL104 | *-* |
|  | 294/13 | 145 | In237-like | + | *-* | *-* | OL104 | IncFIA (HI1) |
|  | 1544/13 | 145 | In237-like | + | *-* | *-* | OL104 | - |
|  | 4464/13 | 145 | In237-like | + | *+* | *-* | OL104 | *-* |
|  | 4806/13 | 145 | In237-like | + | *+* | *-* | OL104 | *-* |
|  | 124/14 | 145 | In237-like | + | *+* | *-* | OL104 | IncM2 |
|  | 626/14 | 145 | In237-like | + | *-* | *-* | OL104 | *-* |
|  | 815/14 | 145 | In237-like | + | *-* | *-* | OL104 | *-* |
|  | 972/14 | 145 | In237-like | + | *-* | *-* | OL104 | *-* |
|  | 1362/14 | 145 | In237-like | + | *+* | *-* | OL104 | *-* |
|  | 3325/14 | 145 | In237-like | + | *-* | *-* | OL104 | *-* |
|  | 85/15 | 145 | In237-like | + | *+* | *-* | OL104 | *-* |
|  | 601/15 | 145 | In237-like | + | *-* | *-* | OL104 | *-* |
|  | 1879/15 | 145 | In237-like | + | *-* | *-* | OL104 | IncpKPC-CAV1321 |
|  | 2641/15 | 145 | In237-like | + | *+* | *-* | OL104 | IncpKPC-CAV1321 |
|  | 4116/15 | 145 | In237-like | + | *-* | *-* | OL104 | *-* |
|  | 4566/15 | 145 | In237-like | + | *-* | *-* | OL104 | *-* |
|  | 347/16 | 145 | In237-like | + | *-* | *-* | OL104 | *-* |
|  | 723/16 | 145 | In237-like | + | *-* | *-* | OL104 | *-* |
|  | 1231/16 | 145 | In237-like | + | *-* | *-* | OL104 | IncM2 |
|  | 2260/16 | 145 | In237-like | + | *-* | *-* | OL104 | *-* |
|  | 2579/16 | 145 | In237-like | + | *-* | *-* | OL104 | *-* |
|  | 2672/16 | 145 | In237-like | + | *+* | *-* | OL104 | *­-* |
|  | 4025/16 | 145 | In237-like | + | *-* | *-* | OL104 | IncFIB_K_ |
|  | 5491/16 | 145 | In237-like | + | *-* | *-* | OL104 | Col (pHAD28), IncFIB_K_, IncFII_K_, IncM2 |
|  | 6209/16 | 145 | In237-like | + | *-* | *-* | OL104 | *-* |
|  | 6428/16 | 145 | In237-like | + | *+* | *-* | OL104 | *­-* |
|  | 6695/16 | 145 | In237-like | + | *+* | *-* | OL104 | IncFIB (pNDM-Mar), IncHI1B (pNDM-Mar), IncM2 |
|  | 7212/16 | 145 | In237-like | + | *-* | *-* | OL104 | IncFIB_K_, IncFII_K_ |
|  | 196/17 | 145 | In237-like | + | *+* | *-* | OL104 | *­*IncM2 |
|  | 460/17 | 145 | In237-like | + | *+* | *-* | OL104 | *­-* |
|  | 905/17 | 145 | In237-like | + | + | - | OL104 | IncN, IncpKPC-CAV1321 |
|  | 1323/17 | 145 | In237-like | + | *+* | *-* | OL104 | *­-* |
|  | 3362/17 | 145 | In237-like | + | *+* | *-* | OL104 | IncM2, IncpKPC-CAV1321 |
|  | 4062/17 | 145 | In237-like | *+* | *-* | *-* | OL104 | IncFIB_K_, IncQ2 |
|  | 4759/17 | 145 | In237-like | *+* | *-* | *-* | OL104 | IncFIB_K_, IncFII_K_ |
|  | 4793/17 | 145 | In237-like | *+* | *-* | *-* | OL104 | IncFII_K_, IncFIBK, IncHI1A (NDM-CIT), IncHI1B (pNDM-CIT) |
|  | 4794/17 | 145 | In237-like | *+* | *-* | *-* | OL104 | IncFII(K), IncFIBK, IncHI1A (NDM-CIT), IncHI1B (pNDM-CIT) |
|  | 5138/17 | 145 | In237-like | *+* | *-* | *-* | OL104 | IncFIB_K_, IncFII_K_ |
|  | 5947/17 | 145 | In237-like | *+* | *-* | *-* | OL104 | IncM2 |
|  | 6676/17 | 145 | In237-like | + | *-* | *-* | OL104 | *­-* |
|  | 6751/17 | 145 | In237-like | + | *-* | *-* | OL104 | *­-* |
|  | 939/18 | 145 | In237-like | + | *-* | *-* | OL104 | *­-* |
|  | 2383/18 | 145 | In237-like | *+* | *-* | *-* | OL104 | IncFIB_K_, IncFII_K_ |
|  | 2437/18 | 145 | In237-like | *+* | *-* | *-* | OL104 | IncFIB_K_, IncFII_K_, IncHI2, IncHI2A |
|  | 5438/18 | 145 | In237-like | + | *+* | *-* | OL104 | *-* |
|  | 5439/18 | 145 | In237-like | *+* | *-* | *-* | OL104 | IncFIB_K_, IncFII_K_ |
|  | 6107/18 | 145 | In237-like | *+* | *-* | *-* | OL104 | *-* |
|  | 7009/18 | 145 | In237-like | + | *+* | *-* | OL104 | *-* |
|  | 7702/18 | 145 | In237-like | *+* | *-* | *-* | OL104 | IncFIB_K_, IncFII_K_ |
|  | 7904/18 | 145 | In237-like | + | *+* | *-* | OL104 | IncFIB_K_ |
|  | 8748/18 | 145 | In237-like | + | *+* | *-* | OL104 | IncFIB_K_ |
|  | 221/19 | 145 | In237-like | *+* | *-* | *-* | OL104 | IncFIB_K_ |
|  | 1636/19 | 145 | In237-like | *+* | *-* | *-* | OL104 | IncFIB_K_, IncFII_K_ |
|  | 2226/19 | 145 | In237-like | + | *+* | *-* | OL104 | IncpKPC-CAV1321 |
|  | 3634/19 | 145 | In237-like | + | *+* | *-* | OL104 | *-* |
|  | 6337/19 | 145 | In237-like | + | *+* | *-* | OL104 | IncFIB_K_, IncM2 |
|  | 6955/19 | 145 | In237-like | *+* | *-* | *-* | OL104 | IncFIB_K_, IncFII_K_ |
|  | 7512/19 | 145 | In237-like | *+* | *-* | *-* | OL104 | IncFIB_K_, IncFII_K_ |
|  | 8138/19 | 145 | In237-like | + | *+* | *-* | OL104 | IncFIB_K_ |
|  | 2990/17 | 145 | In238 | + | *+* | *-* | OL104 | IncFIB_K_ (pCAV1099- 114 ), IncM2, IncU |
|  | **253/19** | 145 | In916 | *+* | *-* | *-* | OL104 | IncA, IncFIBK |
|  | 768/19 | 145 | In916 | + | *+* | *-* | OL104 | IncA, IncFIBK |
|  | 8015/19 | 145 | In916 | + | *+* | *-* | OL104 | IncA, IncFIB_K_ (pCAV1099-114) |
|  | 10246/19 | 145 | In916 | + | *+* | *-* | OL104 | IncA, IncFIB_K_ (pCAV1099-114) |
|  | 1536/13 | 2 | In238-79 | + | + | KL74 | OL104 | IncM1, IncrepA (pKOX) |
|  | 7406/19 | 36 | In916 | + | + | - | O5 | IncA |
|  | 10316/19 | 37 | In71-like | *+* | *-* | *-* | O3/O3a | IncA, IncFIB_K_ (pCAV1099-114), IncN |
|  | 5820/12 | 282 | In238 | + | *+* | *-* | *-* | IncM1 |
|  | 2978/13 | 346 | In916 | + | *+* | *-* | OL104 | IncA |
|  | 5636/16 | 348 | In916 | *+* | *-* | *-* | O5 | IncA, IncFIB_K_ |
|  | 8275/17 | 285 | In916 | + | *+* | *-* | O5 | IncA |
|  | 388/18 | 347 | In916 | + | *+* | *-* | OL104 | IncA |
|  | 10340/19 | 345 | In238 | + | *+* | *-* | O5 | IncFIB (pNDM-Mar), IncHI1B (pNDM-Mar) |
| *K. michiganensis* | 5810/12 | 146 | In238a | *+* | *-* | *-* | O1/O2v1 | IncFIB_K_, IncFII_K_, IncM1 |
|  | 6620/12 | 146 | In238a | *-* | *-* | *-* | O1/O2v1 | IncFIB_K_, IncFII_K_, IncM1 |
|  | 1752/13 | 310 | In41-like | *-* | *-* | KL74 | O1/O2v1 | IncFIB_K_ |
|  | 3664/13 | 310 | In41-like | *-* | *-* | KL74 | O1/O2v1 | IncFIB_K_ |
|  | 3524/15 | 213 | In916 | *+* | *-* | KL152 | O1/O2v1 | IncA |
|  | 1085/17 | 95 | In237-like | *-* | *-* | *-* | O1/O2v1 | IncFIB_K_, IncFII (pKPX1), IncFII_K_, IncU, repB(R1701) |
|  | 1086/17 | 95 | In237-like | *-* | *-* | *-* | O1/O2v1 | IncFIB_K_, IncFII (pKPX1), IncFII_K_, IncU, repB (R1701) |
|  | 10279/19 | 95 | In237-like | *-* | *-* | *-* | O1/O2v1 | IncFIB_K_, IncFII (pKPX1), IncFII_K_, IncU, repB (R1701) |
|  | 4445/19 | 210 | In238 | *-* | *-* | *-* | O1/O2v1 | Col (pHAD28), IncHI1A (NDM-CIT), IncHI1B (NDM-CIT), IncN3 |
|  | 8545/19 | 180 | In916 | *-* | *-* | *-* | O1/O2v1 | IncA, IncU |
|  | 9543/19 | 180 | In916 | *-* | *-* | *-* | O1/O2v1 | IncA, IncU |
| *K. grimontii* | 5535/16 | 172 | In110 | *-* | *+* | *-* | O1/O2v1 | IncHI2, IncHI2A |
| *K. pasteurii* | 6490/18 | 229 | In238a | + | *+* | *-* | O1/O2v1 | IncFIB_K_ (pCAV1099-114), IncHI1B (pNDM-Mar), IncR |
| *K. spallanzanii* | 7090/18 | - | In916 | *-* | *-* | KL24 | *-* | IncA |

**Figure S1.** Geographic distribution and clonal analysis of *K. oxytoca* ST145 in Poland. **A.** Geographic distribution of the isolates shown on the map with main administrative regions. Circles represent medical centres where the isolates were recorded. Sizes of the circles are proportional to numbers of cases. **B.** SNP-based minimum spanning tree of the *K. oxytoca* ST145 isolates. Lengths of branches are related to numbers of SNPs between linked isolates. Numbers of SNPs are indicated above the branches or next to the dots.

**A**

**B**

**Figure S2.** SNP-based phylogenetic tree of Polish *K. oxytoca* ST2 isolate compared with the international ST2 genomes available in RefSeq. Numbers in the inner circle correspond to original numbers of the study isolates or RefSeq assembly numbers. The presence of carbapenemases is indicated in the outer circles using corresponding colors. The country of origin is presented with country codes: AR, Argentina, CH, Switzerland; CN, China; DE, Germany; ES, Spain; GB, Great Britain; PL, Poland; US, USA. The tree was constructed using Parsnp and visualized with iTOL.


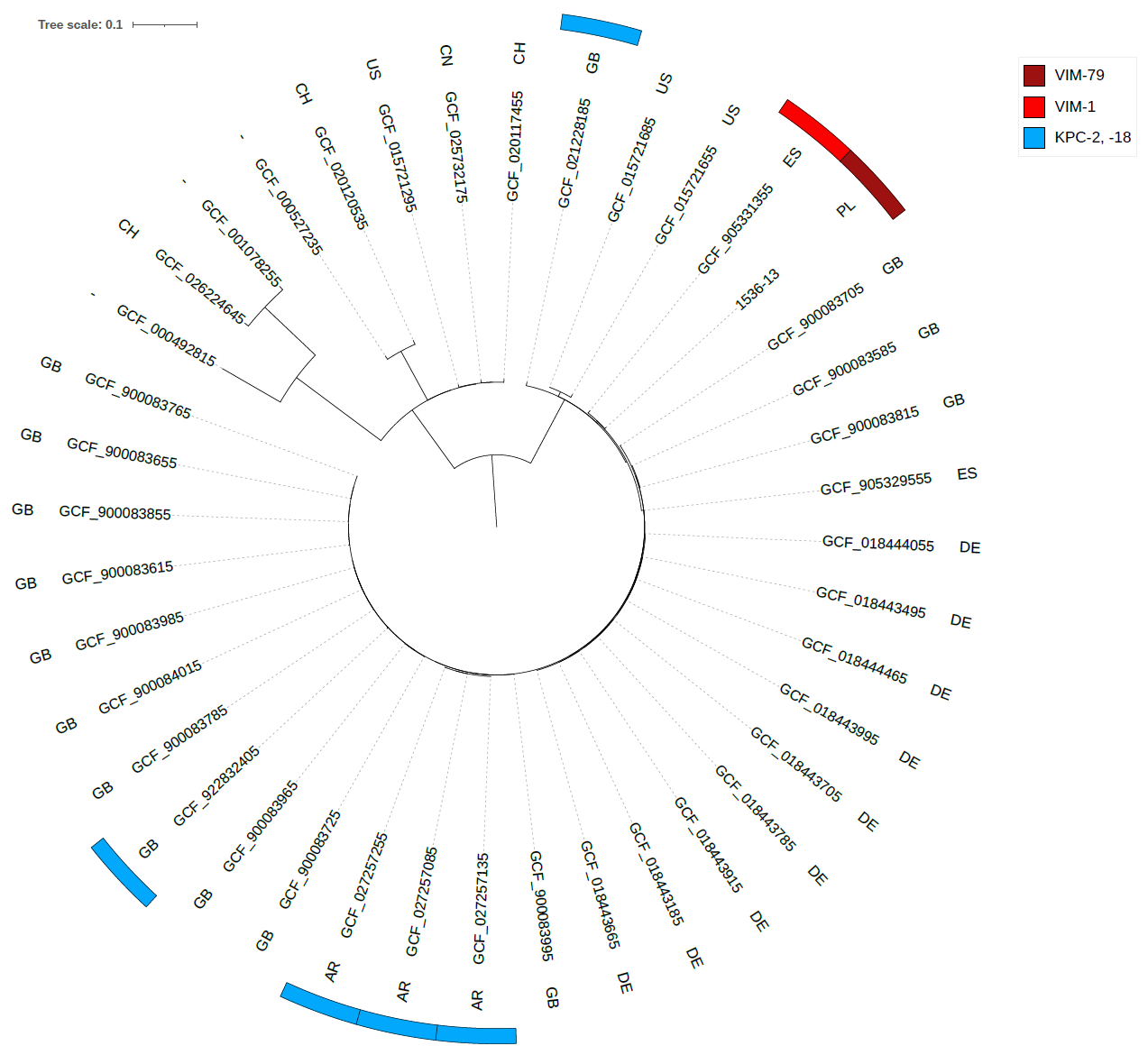


**Figure S3.** Comparison of the VIM-1-encoding (integron In916) IncA p253A plasmid to previously reported plasmids of the highest homology: p743A (Poland, OQ111274; inner, thin black circle), p5955A (Poland; OQ111275), p7753A (Poland, OQ111276), p9546_2 (Poland; ONO081626), p550_IncA_VIM (Italy; CP058224), PRIVM0001_VIM-1 (The Netherlands; MH220284), pFDL-VIM (Italy; MN783744), pKC-BO-N1_VIM (Italy; MG228427) and pGA_VIM (Italy; MN783743). The outer rings refers to the annotation of p743A, with the selected genes indicated. The percentage of sequence identity is reflected by color intensity. The picture was created using BRIG software


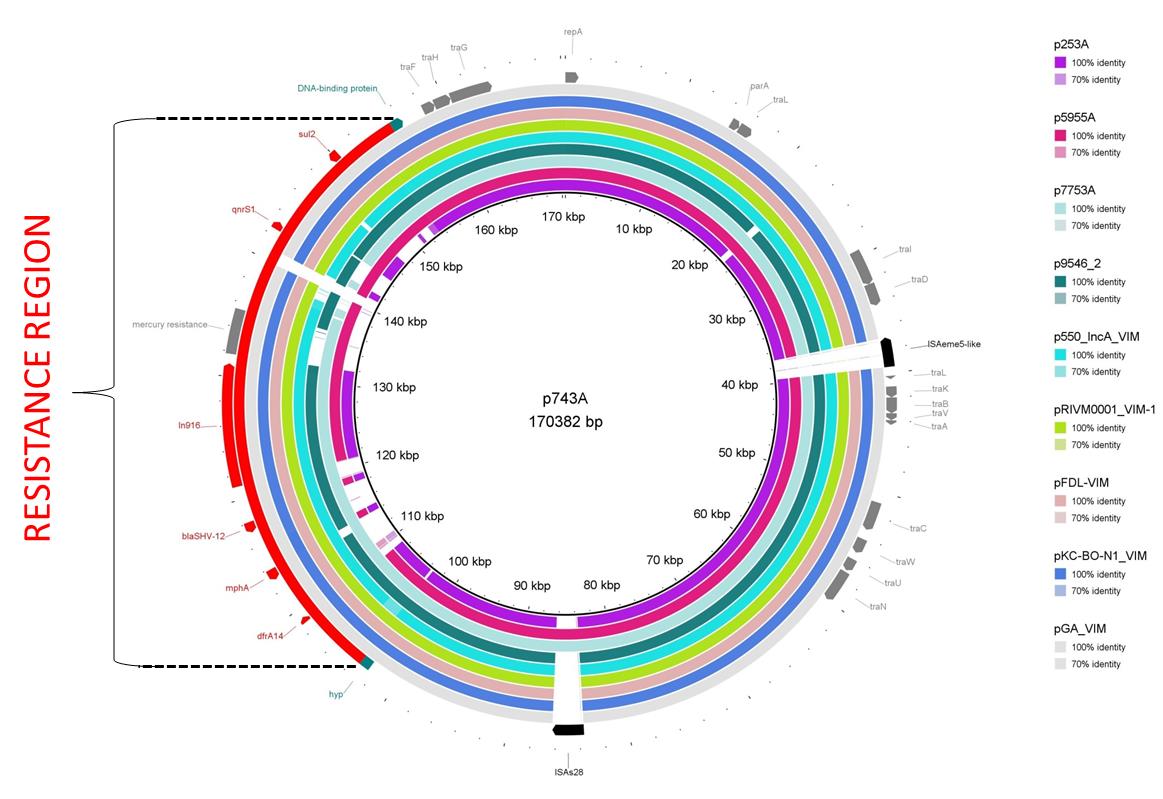


**Figure S4.** Comparison of the AMR region of the In916-carrying IncA-like plasmid p253A with the corresponding part of p5955A (Poland, *E. hormaechei*, OQ111275), p9546/19_2 (Poland, *K. pneumoniae*, ON081626) and p743A (Poland, *E. hormaechei*, OQ111274). The percentage of sequence identity is reflected by the gray color intensity. Individual loci (antibiotic AMR genes, mobile genetic elements and integration sites) are marked by coloured arrows or triangles as explained below. The picture was created using the Easyfig 2.2.5 software
